# Interaction of lipopolysaccharides at intermolecular sites of the periplasmic Lpt transport assembly

**DOI:** 10.1038/s41598-017-10136-0

**Published:** 2017-08-29

**Authors:** Cedric Laguri, Paola Sperandeo, Kevin Pounot, Isabel Ayala, Alba Silipo, Catherine M. Bougault, Antonio Molinaro, Alessandra Polissi, Jean-Pierre Simorre

**Affiliations:** 1 Université Grenoble Alpes, Institut de Biologie Structurale, 71 avenue des Martyrs – CS10090, 38044 Grenoble cedex 9, France; 2 CEA, DSV, Institut de Biologie Structurale, 71 avenue des Martyrs – CS10090, 38044 Grenoble cedex 9, France; 3 CNRS, Institut de Biologie Structurale, 71 avenue des Martyrs – CS10090, 38044 Grenoble cedex 9, France; 4 University of Milano, Department of Pharmacological and Biomolecular Sciences, Via Balzaretti 9, Milano, Italy; 5 University of Naples Federico II, Department of Chemical Sciences, via cinthia 4, Napoli, Italy

## Abstract

Transport of lipopolysaccharides (LPS) to the surface of the outer membrane is essential for viability of Gram-negative bacteria. Periplasmic LptC and LptA proteins of the LPS transport system (Lpt) are responsible for LPS transfer between the Lpt inner and outer membrane complexes. Here, using a monomeric *E. coli* LptA mutant, we first show *in vivo* that a stable LptA oligomeric form is not strictly essential for bacteria. The LptC-LptA complex was characterized by a combination of SAXS and NMR methods and a low resolution model of the complex was determined. We were then able to observe interaction of LPS with LptC, the monomeric LptA mutant as well as with the LptC-LptA complex. A LptC-LPS complex was built based on NMR data in which the lipid moiety of the LPS is buried at the interface of the two β-jellyrolls of the LptC dimer. The selectivity of LPS for this intermolecular surface and the observation of such cavities at homo- or heteromolecular interfaces in LptC and LptA suggests that intermolecular sites are essential for binding LPS during its transport.

## Introduction

**Figure 1:**
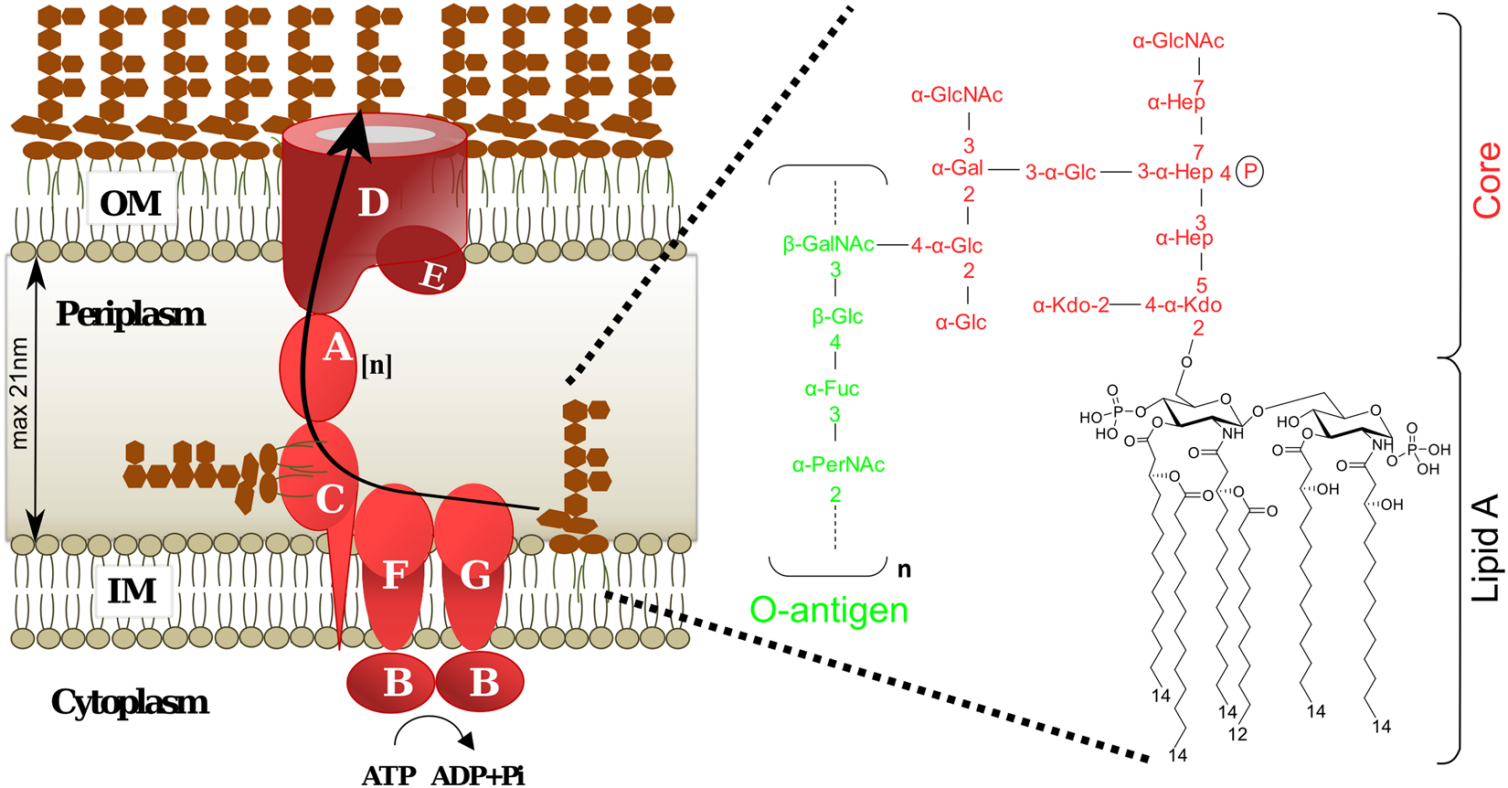
Export of LPS to the cell surface. The Lpt molecular machine (LptA-G) transports LPS across the periplasm and inserts it into the outer membrane (OM) at the expense of ATP hydrolysis (left). The number [n] of LptA molecules required to cross the 210-Å-wide periplasm^30^ is not determined experimentally. The LptDE complex finally inserts the LPS into the OM. On the right the chemical structure of *E. coli* O157:H7 LPS used in this study^51, 52^.

The cytoplasm of Gram-negative bacteria is typically surrounded by two membranes with different lipid composition that are separated by an aqueous compartment called the periplasm^1^. While the inner membrane (IM) is almost exclusively made of phospholipids, the outer membrane (OM) is highly asymmetric and contains phospholipids in its inner leaflet and an unusual glycolipid, lipopolysaccharide (LPS), in its outer leaflet. The LPS molecule is made of three covalently linked moieties: the lipid A, which is the hydrophobic anchor in the membrane, a core oligosaccharide, and a variable O-antigen sugar chain^2^ (Fig. 1). The presence of the tightly packed LPS layer at the outer leaflet makes the OM fairly impermeable and protects Gram-negative bacteria from harmful compounds such as detergents and lipophilic antibiotics^3^.

In *Escherichia coli* the LPS molecules are synthesised in the cytoplasm and at the IM, flipped across the IM by the dedicated ABC transporter MsbA and then exported to the OM by the Lpt molecular machine^4^. This machinery is made up of seven proteins (LptA-G) that physically interact and assemble in a complex spanning the entire envelope^5^ (Fig. 1). The Lpt machinery is organized in two subassemblies^6^. At the IM the LptB_2_FG complex, associated to the bitopic protein LptC, constitutes an unusual ABC transporter that utilizes ATP hydrolysis to promote the transport of LPS across the periplasm^7–12^. At the OM the LptDE proteins constitute the translocon that delivers the LPS to the cell surface^13–15^. LptA is a key protein that connects the LptB_2_CFG complex to the LptDE translocon by interacting with LptC and the periplasmic region of LptD via its N- and C-terminal ends, respectively^16, 17^. The crystal structures of all (LptA, LptBFG, LptC, and LptDE) Lpt proteins are known^12, 18–20^. Notably, Lpt proteins with periplasmic domains (LptA, LptC, LptF, LptG and LptD) share a very similar β–jellyroll architecture. Based on structural and photo-crosslinking studies LptC, LptA and the N-terminal region of LptD are proposed to interact via their homologous β–jellyroll domains to form a periplasmic bridge connecting IM and OM^16, 21^. This protein bridge, constituted by one LptC, one LptD and a multimer of LptA of unknown length, is in agreement with the LptA tendency to oligomerize in solution^22, 23^. Indeed, mutagenesis and X-ray studies suggest that *in vitro* LptA can interact with itself through its N- and C-termini to form a head to tail multimeric assembly^18^. *In vivo* photo-crosslinking studies suggest that the β–jellyroll folds of the proteins in the periplasmic bridge build up a continuous hydrophobic groove able to accommodate the lipid moiety of LPS during its journey across the periplasm^16, 17^. Based on these data Kahne and co-workers proposed the so called PEZ model of LPS transport to the cell surface^21^. According to this model the hydrolysis of ATP is used to provide the energy to extract LPS from the IM and to push it along the continuous groove formed by LptC, LptA and the N-terminal region of LptD. Then LPS is delivered to the LptDE translocon for its direct insertion in the outer leaflet of the OM.

Despite the availability of a wealth of structural and functional data many important details are still missing for both the architecture of the Lpt machinery and the mechanism of LPS transport. Molecular level data are more specifically needed on how complexes assemble, bind LPS and transport it through the periplasm. Gaining such experimental information on LPS interaction with proteins of the Lpt machinery is particularly challenging due to the heterogeneity of LPS molecules, as well as their high hydrophobicity. Deciphering the protein interfaces remains also a crucial step in order to be able to propose molecules that could target the Lpt interactome and block LPS transport. Inhibitors of LPS biogenesis could perform as effective antibiotics, either alone or in combination with known drugs, since improper LPS assembly creates a defective OM. Indeed, a peptidomimetic antibiotic specifically targeting LptD, an OM component of the Lpt machinery, has been recently identified in a high throughput screen against *Pseudomonas sp*.^24, 25^.

Here, we report the first description of the LptC-LptA complex at the atomic level as well as the molecular details of the interaction of LPS with the LptC and LptA systems. Importantly, we found that LPS is accommodated in cavities that are formed at Lpt protein-protein interfaces thus suggesting that these itermolecular cavities are essential for the transfer process. Moreover, by modifying the LptA multimeric interface, we show that an LptA oligomeric form is not strictly essential for *E. coli* survival and growth.

## Results

### Monomeric LptA interacts with LptC and is functional to transport LPS

LptA has a high propensity to oligomerize in solution^22, 23^, through establishment of a continuous β-sheet from N and C-terminal B-strands of two LptA monomers. We thus designed a truncated version of LptA at its C-terminus part (Δ_160-185_). This protein, termed LptA_m_, lacks the last C-terminal β-sheet creating a possible disruption of the LptA-LptA interface.

**Figure 2:**
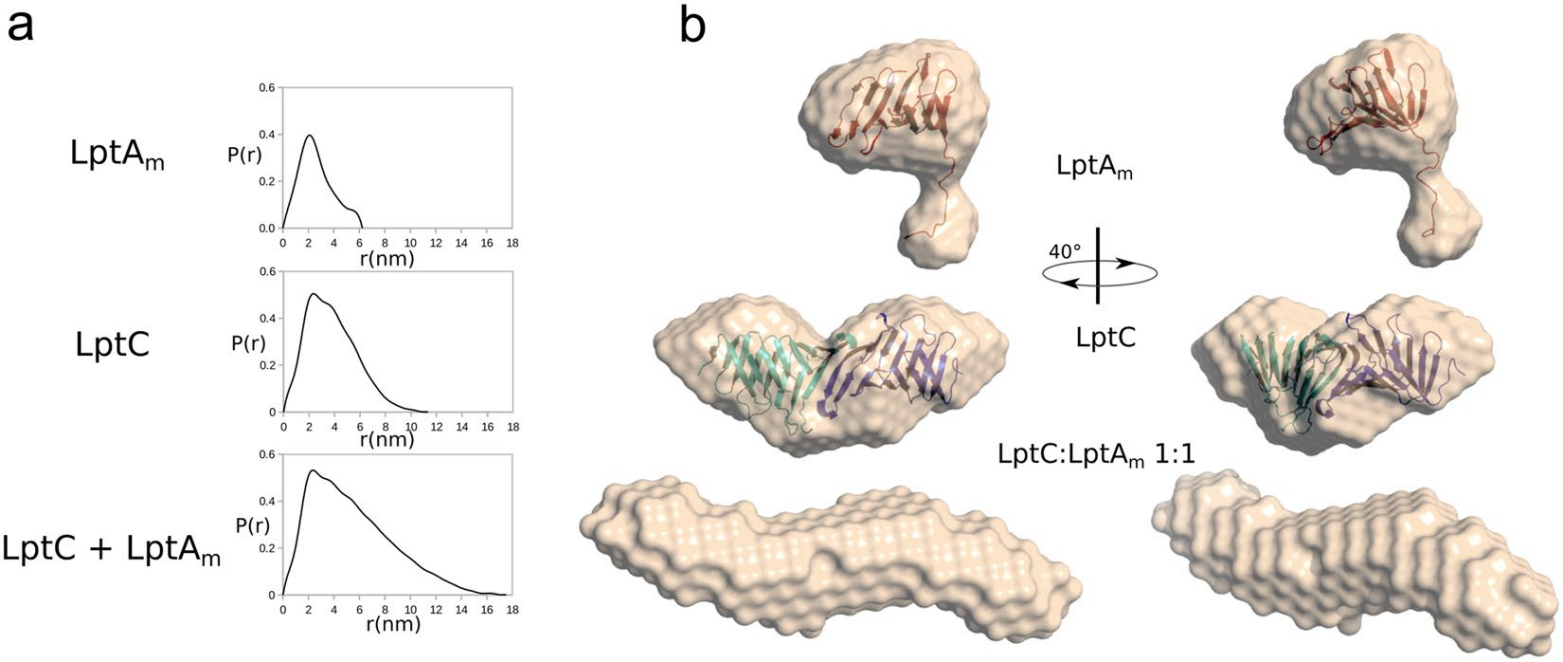
LptA_m_ is momomeric and forms a complex with LptC. (**a**) SAXS analysis of LptA_m_, LptC and LptC:LptA_m_ mixture. P(r) distribution plot is calculated from the X-ray scattering curves of LptA_m_, LptC and of a LptC:LptA_m_ mixture in a 1:1 ratio. The estimated Rg as well as maximum interatomic distance (D_max_) increase for the LptC-LptA mixture confirming the formation of a complex. (**b**) SAXS *Ab initio* envelope determination of LptA_m_, LptC with their fitted protein structure (see SAXS section in Methods for atomistic models used) inside as ribbons, and LptC-LptA_m_ complex calculated with Damfilt.

Biophysical characterization of LptA_m_ by SEC-MALLS (Size Exclusion Chromatography Multiple Angle Laser Light Scattering) and Small Angle X-ray Scattering (SAXS) confirms that LptA_m_ is monomeric with an estimated molecular weight of 15 kDa (Fig. 2a, Figure S1 and Table S1). In parallel, intensity curves measured by SAXS on LptA_m_ gives a distance distribution with a D_max_ of 6.24 nm and a Rg of 2.08 nm in agreement with a monomeric form of the protein. Figure 2b shows the low resolution bead model fitted by the Dammif/Damfilt softwares from the SAXS curves for LptA_m_ with a superimposition of the atomistic structure of a monomer of LptA extracted from the oligomeric X-ray structure (PDB 2R1A chain B see SAXS section in Methods).

Since the solution study of LptA alone and of the LptC-LptA complex is hindered by the oligomerisation tendency of LptA, the LptA_m_ protein represents an ideal molecular tool to investigate the different interactions involved during the transport of the LPS through the hydrophilic periplasm. Before studying the LptC-LptA_m_ complex, an LptC construct lacking the 23 residues long N-terminal transmembrane domain was characterized by SEC-MALLS and SAXS (Fig. 2a and b). Notably, this LptC construct is functional as, when exported to the periplasm, it can assemble an Lpt machinery fully proficient in LPS transport^17^. Our results indicate that this construct forms a stable dimer in solution, with the SAXS calculated envelope of LptC fitting with the symmetrical head-to head dimer obtained by X-ray crystallography^17, 19^. To investigate the possibility of forming an LptC-LptA_m_ complex, SAXS data were then recorded on an LptC:LptA_m_ 1:1 mixture (Fig. 2, Figure S1 and Table S1). The presence of an LptC-LptA_m_ complex is characterized by an increase in the estimated Rg (4.4 vs 3.0 nm) and D_max_ (17.5 vs 11.3 nm), compared to LptC alone. The bead model, fitted from the SAXS curve, shows a longer shape when compared to the bead model obtained for LptC alone (Fig. 2b). These data suggest the formation of a dimer of LptC interacting with LptA_m_.

**Figure 3:**
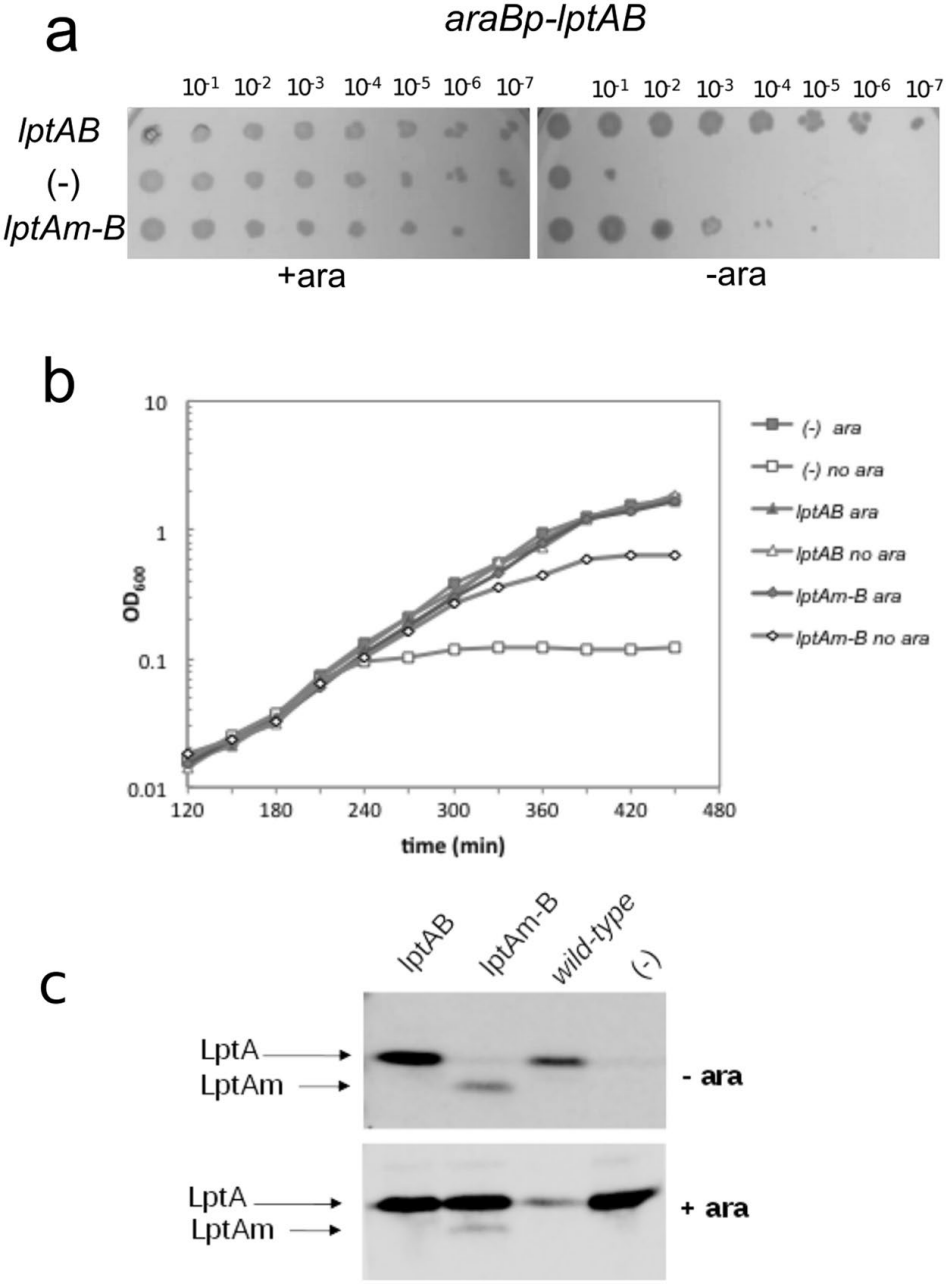
LptA momomeric mutant (LptA_m_) is functional *in vivo*. The *araBp-lptAB* arabinose dependent conditional mutant in which the chromosomal *lptAB* genes are under the control of the arabinose dependent *araBp* promoter, is complemented by the *lptA*_*m*_ *-lptB* allele. The steady-state levels of LptA and LptAm are analyzed by Western blotting. (**a**) *araBp-lptAB* cells expressing wild type and mutant *lptA*_*m*_ alleles were grown in LD with 0.2% arabinose and 100 μg/ml ampicillin. Serial dilutions in microtiter plates were replica plated on the same medium with (+ara) or without (−ara) arabinose. Serial dilutions are indicated on the top of the panel. (−): empty vector. (**b**) *araBp-lptAB* cells expressing wild type and mutant *lptA*_*m*_ alleles grown in LD with 0.2% arabinose and 100 μg/ml ampicillin up to an optical density at 600 nm (OD_600_) of 0.2, were diluted 350-fold in fresh medium (supplemented with ampicillin), with (ara) or without (no ara) 0.2% arabinose. Growth was monitored by measuring OD_600_. (**c**) *araBp-lptAB* cells bearing plasmids expressing *lptAB*, *lptA*_*m*_ *-B* and empty vector (−) were grown with (+ara) or without (−ara) arabinose as described in panel B and samples were collected at 300 min. The AM604 (wild type) strain was grown in LD up to OD_600_ of 0.6. Samples were analyzed by Western blotting using anti-LptA antibodies. Equal amount of cells (0.24 OD_600_ units) were loaded onto each lane.

Since LptA_m_ can interact with LptC, we have tested whether LPS transport can be accomplished by Lpt machines carrying C-terminally truncated LptA. LptA_m_ can support growth of the arabinose dependent *araBp*-*lptAB* conditional mutant^8^, under non-permissive conditions (no arabinose) although with a lower efficiency if compared to the wild type protein (Fig. 3a and b). We then assessed under the same conditions the steady state level of LptA_m_ and found that it was lower than that of wild type LptA expressed from the complementing plasmid. Importantly, when the conditional *araBp*-*lptAB* mutant ectopically expressing LptA_m_ was grown without arabinose, the level of LptA_m_ was even lower than that of chromosomally expressed LptA in the wild type strain (Fig. 3c). Overall these results suggest that a monomeric form of LptA can, at least in part, fulfill its role in the transport of LPS to the OM.

### NMR characterization of the complexes between functional LptA_m_ and LptC

**Figure 4:**
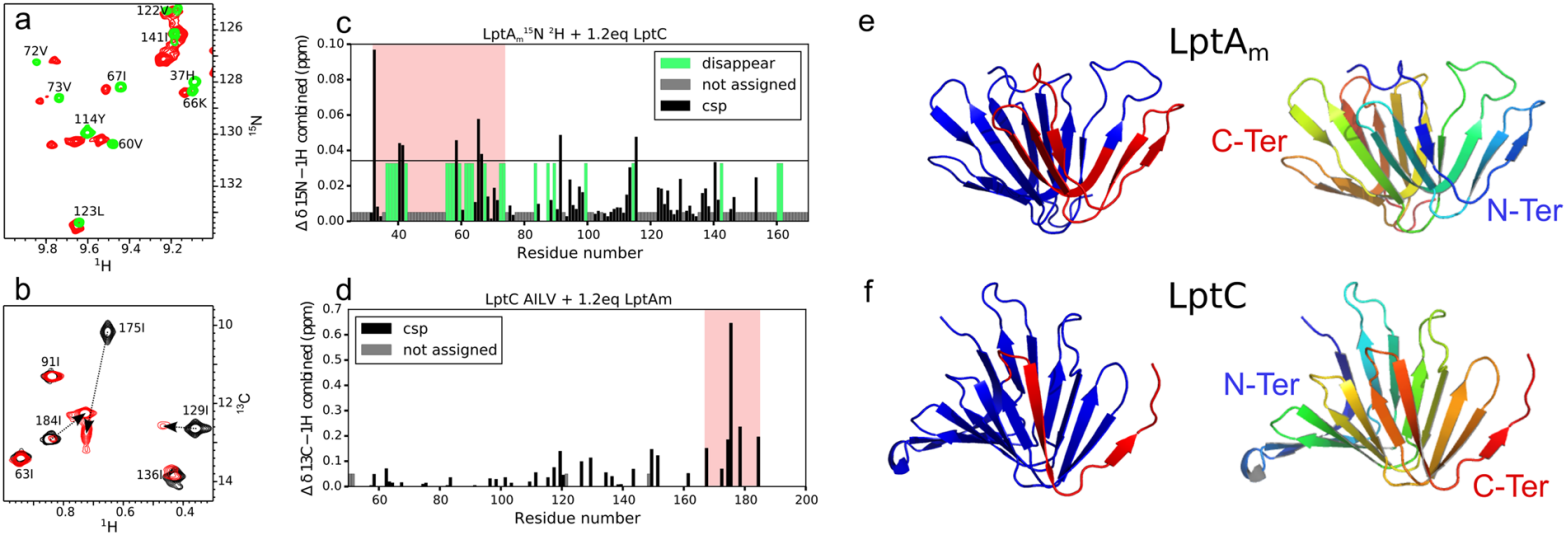
LptC-LptA_m_ interaction by NMR spectroscopy. (**a**) [^1^H, ^15^N]-correlation spectrum of [^2^H, ^15^N]-labeled LptA_m_ in absence (green) and presence (red) of 1.2 molar equivalent of [^1^H, ^14^N]-LptC. (**b**) [^1^H, ^13^C]-correlation spectrum of LptC specifically ^13^C labeled and protonated on A^β^I^δ1^L^δ1^V^γ1^ methyl groups in absence (black) and presence (red) of 1.2 equivalent [^1^H, ^12^C]-LptA_m_. (**c**) and (**d**) Chemical shift perturbation induced upon complex formation on LptA_m_ and LptC proteins, respectively. Two regions of LptA_m_ and LptC are perturbed upon complex formation (light red boxes), the N-terminus and C-terminus of the proteins, respectively. These regions are represented in red on the ribbon structures of (**e**) LptA_m_ (residues 29–159) and (**f**) LptC (residues 56–185). The right panels in (e) and (f) show LtpA_m_ and LptC in the same orientation as in the left panel with a blue-to-red gradient from N- to C-termini of the proteins.

To further investigate the molecular structure of the LptC-LptA_m_ complex observed by SAXS, NMR experiments were performed using [^2^H, ^13^C, ^15^N]-fully labeled samples or [^2^H, ^12^C, ^15^N]-samples specifically ^13^C-labeled and protonated on A^β^I^δ1^L^δ1^V^γ1^ methyl groups (Figure S2). NMR study of high molecular weight macromolecules is limited by overlaps and broadening of signals, and necessitates isotopic labeling. NMR active nuclei are introduced either homogeneously (^2^H, ^13^C, ^15^N) or specifically (^13^C-^1^H methyls) with defined *E. coli* growth media (see methods section). They constitute probes which provide atomic resolution information on defined areas of the macromolecules observed. Assignments of the backbone and methyl NMR resonances were obtained for LptA_m_ and LptC using a set of heteronuclear experiments (See methods section). To identify the residues involved in the interface between LptC and LptA_m_, 2D-[^1^H, ^15^N]-BEST-TROSY-HSQC spectra were recorded on [^2^H, ^13^C, ^15^N]-LptC-[^1^H, ^12^C, ^14^N]-LptA_m_, [^1^H, ^12^C, ^14^N]-LptC-[^2^H, ^13^C, ^15^N]-LptA_m_. These spectra show amide NH signals of the ^15^N labeled protein in presence and absence of the unlabeled partner (Fig. 4a). Due to the size of the complex and to improve the resolution, specific labeling was used and methyl selective 2D-[^1^H, ^1^C]-BEST-TROSY-HMQC were recorded on [^2^H, ^15^N, ^1^H/^13^C-(A^β^I^δ1^L^δ1^V^γ1^)]-LptC-[^1^H, ^12^C, ^14^N]-LptA_m_. Here specific methyl groups of four aminoacids (AILV) of LptC are observed in presence and absence of unlabeled LptA_m_. Formation of a complex is characterised by changes in the chemical shift (resonance frequency) of nuclei at the vicinity of the binding site termed CSP (chemical shift perturbation). All NMR experiments were recorded at submillimolar concentrations and showed total complex formation, and total disappearance of uncomplexed species signals, at an approximate molar ratio of 1-to-1 in LptC-and LptA_m_. Since LptC is stabilized in a dimeric form, the NMR data support the formation of a LptC-LptA_m_ 2:2 complex. When LptC-LptA_m_ complexes are subjected to SEC-MALLS analysis, dilution during the course of the SEC allows complexes to dissociate(Figure S1). Even in a presence of a two-times molar excess of LptA_m_, we observe a complex of 54-kDa, not fully assembled that corresponds to a 2:1 LptC-LptA_m_ stoechiometry. LptC and LptA_m_ do not form a highly stable complex and partly dissociate in the course of the SEC, consistent with the estimated LptC-LptA Kd of ~4 µM^26^.

Comparison of the LptC or LptA_m_ resonances chemical shift in the free protein forms and in the LptC-LptA_m_ complex (Fig. 4a and b) reveals the residues that are located in the close proximity of the interacting protein surfaces. Chemical shift perturbations (CSP) observed in the LptC-LptA_m_ complex are displayed in Fig. 4c and d for LptA_m_ HN and LptC methyl-HC resonances, respectively. A largely perturbed region, with CSP larger than two standard deviations and disappearing residues, is identified between residues T32 and V73 at the N-terminus β-strands of LptA_m_, while methyl groups of residues L167 to I184 at the C-terminus of LptC are largely perturbed as well. These regions are reported on the structure of the individual proteins in Fig. 4e and f, respectively. Altogether these results point out the role of the N-terminus domain of LptA_m_ and the role of the C-terminus domain of LptC in the LptC-LptA_m_ complex formation. The fact that the N-terminus of LptC remains unaltered suggests that the head-to-head dimeric interface is not affected by the presence of LptA_m_, fully consistent with the formation of a 2:2 LptC-LptA_m_ complex.

### Structural determination of the LptC-LptA_m_ complex using SAXS and NMR

**Figure 5:**
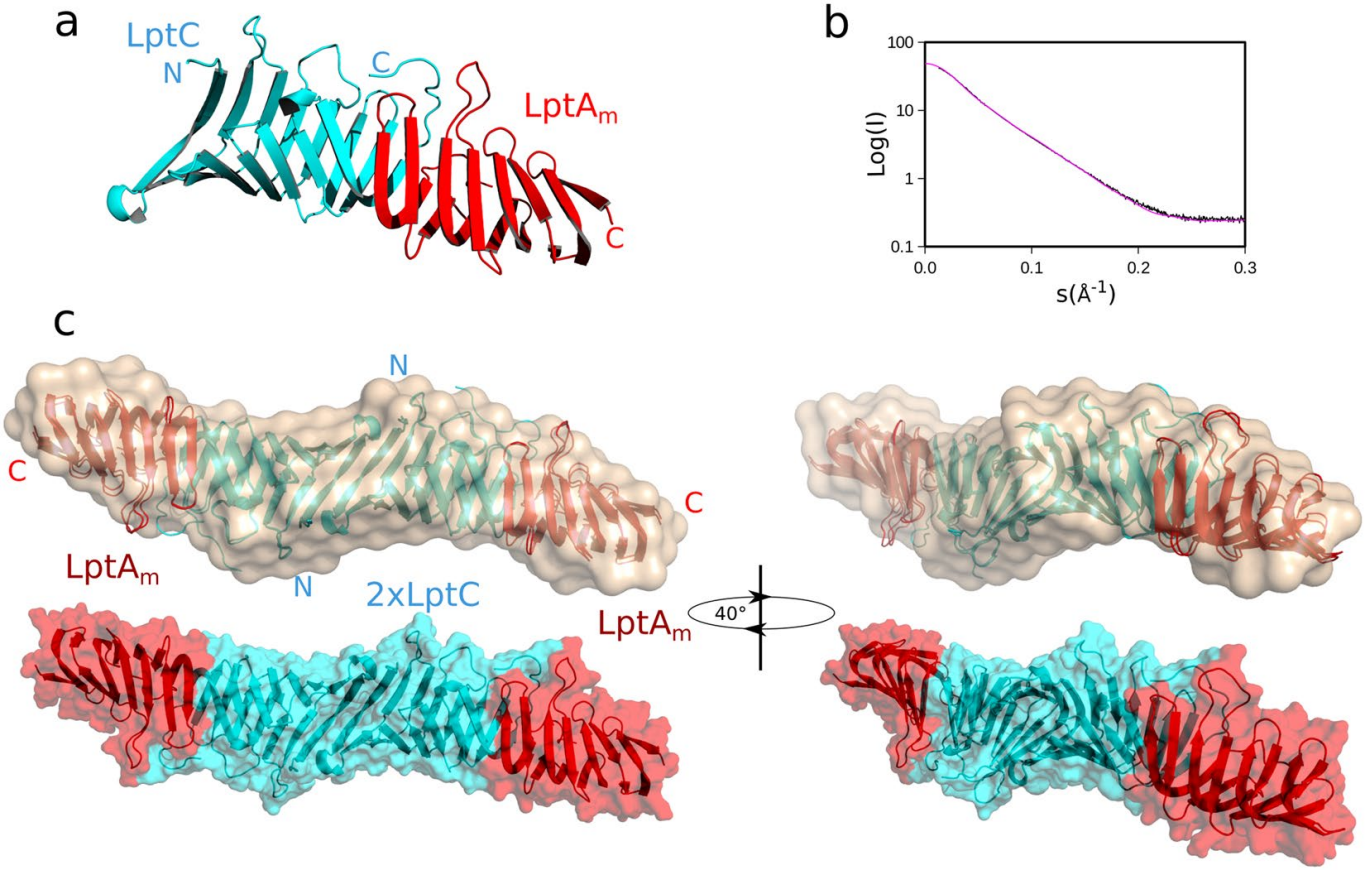
Combined NMR-SAXS model of the LptC-LptA_m_ complex. (**a**) Representation of LptC-LptA_m_ half complex. LptC and LptA_m_ interact mainly through the formation of a continuous antiparallel β-sheet between the C-terminal β-strand of LptC and the N-terminus β-strand of LptA_m_. (**b**) Overlay of the backcalculated SAXS curve from the best energy NMR-SAXS model (magenta) with respect to the raw data (black). The chi-squared value is χ^2^ = 9.25. (**c**) Ribbon representation of the best NMR-SAXS LptC-LptA_m_ complex fitted into the *ab-inito* envelope of the complex (wheat). In the lower panel the best NMR-SAXS structure is represented in cyan-red as a surface for comparison with the upper-panel SAXS envelope. LptC and LptA_m_ molecules are shown in cyan and in red, respectively.

Since SAXS data provides a low resolution global molecular envelope of the LptC-LptA_m_ complex (Fig. 2b) and CSP measured by NMR allows the identification of the intermolecular surfaces (Fig. 4), the two techniques were combined to determine the structure of the LptC-LptA_m_ complex. The docking of the proteins was performed using HADDOCK^27^ protocols. As starting structures of the binding partners, we used the X-ray structure of LptC dimer (PDB 3MY2) and the X-ray structure of the LptA (2R1A chain B) on which extremities and loop residues lacking in the X-ray structures were rebuilt (See SAXS section in Methods). The docking protocol was performed using ambiguous restraints between residues of LptC and LptA that showed significant chemical-shift perturbation in the 2D-[^1^H, ^15^N]- and 2D-[^1^H, ^13^C] correlation experiments. In order to introduce the SAXS data, χ^2^-based SAXS scoring were introduced in the energy calculation of HADDOCK^28^. Based on this recalculated energy, sets of docking models were re-ranked according to their fit to the experimental SAXS (χ^2^) data (Figure S3). The lowest energy structure of the best cluster of solutions for the LptC-LptA_m_ complex is presented in Fig. 5a together with the fit of the SAXS experimental I(s) curve to the experimental data (Fig. 5b) and the fit of the LptC-LptA_m_ complex model structure to the SAXS envelope (Fig. 5c). In the LptC-LptA_m_ complex, LptC precisely occupies the same position as LptA in the LptA oligomer (Figure S4A).

### Molecular interaction of LPS with LptC

In order to observe the formation of a complex between LPS molecules and LptC, [^13^C, ^15^N]-labeled LPS was titrated with unlabeled LptC and its resonances followed by [^13^C, ^1^H]- correlation experiments (Figure S6); *E. coli* (O157: H7) smooth type LPS was used. In presence of LptC, a signal belonging to LPS appears upfield in the methyl spectral region. A similar upfield shift of the methyl group of LPS lipid chains was observed in presence of the CD14 protein^29^ suggesting that the new LPS resonance corresponds to LptC-bound LPS. No other difference could be observed in the LPS NMR spectrum, in particular, no O-antigen signals were shifted.

**Figure 6:**
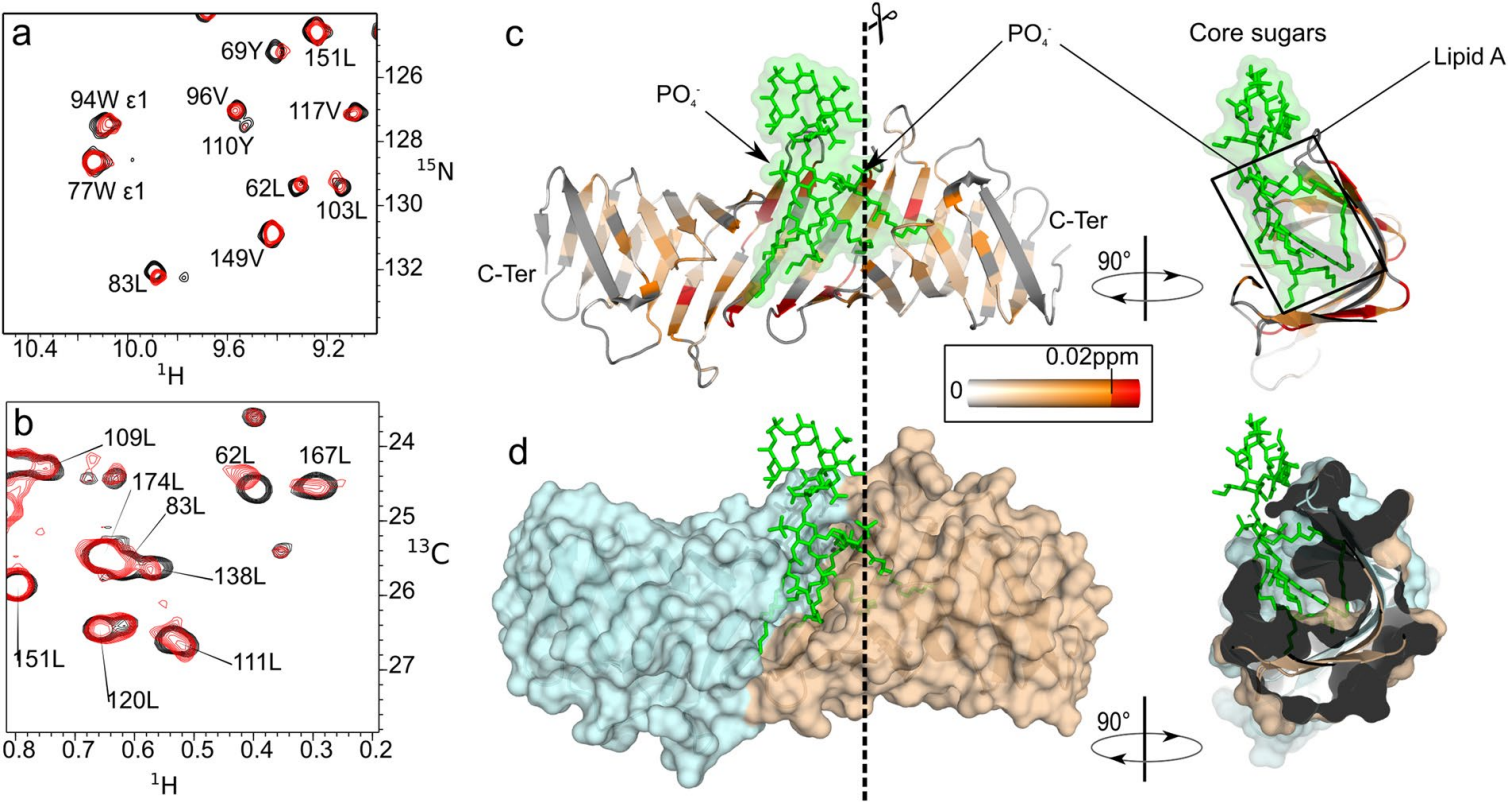
Interaction of LptC with LPS in solution. (**a**) [^1^H, ^15^N]-correlation spectrum of [^2^H, ^15^N]-labeled LptC in presence (red) and absence (black) of 0.8 mg/ml of LPS. (**b**) [^1^H, ^13^C]-correlation spectrum of [^2^H, ^15^N, ^1^H/^13^C-(A^β^I^δ1^L^δ1^V^γ1^)]-labeled LptC in presence (red) and absence (black) of 0.8 mg/ml of LPS. (**c**) Combined ^1^H and ^15^N CSP observed on LptC upon LPS binding are displayed on a ribbon representation of the best HADDOCK LptC-LPS model calculated from these CSP. Residues with a CSP value superior to two standard deviations (0.02 ppm) are displayed in red and residues with CSP values below this threshold are colored using an orange-to-white gradient color-code. (**d**) Surface representation of the best HADDOCK LptC-LPS model presented with the same orientation as in (**c**). The two LptC molecules are colored differently to visualize the localization of the intermolecular interface and cavity, where LPS binds.

In order to define the site of interaction of LPS on the LptC dimer, [^1^H, ^15^N]-BEST-TROSY-HSQC spectra on [^2^H, ^13^C, ^15^N]-LptC and [^1^H, ^13^C]-BEST-TROSY-HMQC on [^2^H, ^15^N, ^1^H/^13^C-(A^β^I^δ1^L^δ1^V^γ1^)]-LptC were recorded before and after addition of LPS molecules at 0.8 mg/ml. LPS induce chemical shift variations on both amide and methyl resonances of LptC (Fig. 6a,b and Figure S5). LptC cannot be saturated with LPS as LPS solubility in water is very low and limits the concentration of molecules accessible for binding. The small but significant NMR CSP for the complex are reported on the structure of the LptC dimer 2 (Fig. 6c). The majority of the CSP are observed at the N-terminus of the LptC head-to-head dimer around the cavity formed at the dimeric interface.

The CSP information determined from LPS binding on LptC identifies a well defined interaction interface: LPS binds in the cavity formed around the dimer interface (Fig. 6c). Furthermore methyl group(s) of the fatty acids of LPS molecules are involved in the LPS/LptC surface interaction (Figure S6). This information was thus used to obtain a LptC-LPS structural model with HADDOCK. Since only LPS resonances related to lipid A moiety were affected by the presence of LptC, structure calculation was performed in the absence of O-antigen on the LPS molecule construct. CSP data on both methyl and amide protons of LptC were entered as ambiguous restraints to any atom of the lipid A or core sugar part of the LPS molecule. The structure with the lowest energy obtained from this calculation is represented in Fig. 6c and d. LPS lipid A acyl chains are found buried in the LptC dimeric cavity and cause little structural changes in the initial LptC dimeric structure with an RMSD on backbone Cα of 0.9 Å, with respect to the apo-molecule. While precise definition of the LPS molecule inside LptC cannot be accurately determined, the location of the phosphate groups of LPS is pivotal for LptC-LPS interaction. In fact, several residues, some of which highly conserved in LptC from different bacteria, are in position to make contacts with the phosphate backbone (N/Q105, and R/K107 in particular) (Figure S7).

### Molecular interaction of LPS with the LptC-LptA_m_ complex

**Figure 7:**
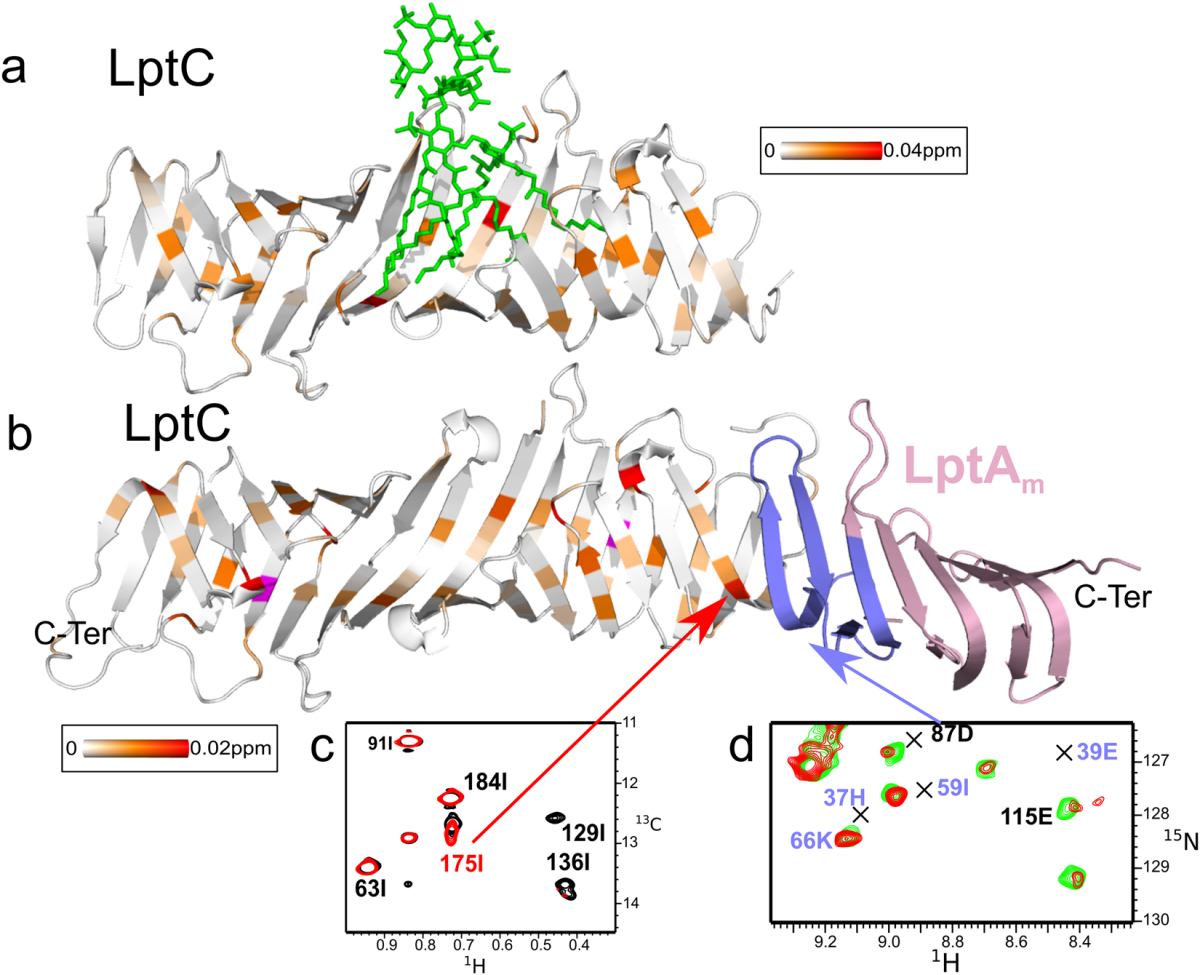
Interaction of LptC dimer and of LptC-LptA_m_ complex with the *E. coli* lipopolysaccharide. Representation of methyl ^1^H and ^13^C combined CSP induced by LPS (0.8 mg/ml) in the isolated LptC dimer (**a**) or in the LptC-LptA_m_ complex (**b**). Gradient colors used for the display of the CSP values are shown. Residue in magenta disappeared upon LPS addition. For clarity only one LptA_m_ molecule of the LptC-LptA_m_ complex is represented. Residues of LptA_m_ colored in blue correspond to the perturbed area upon LptC interaction (as in red on Fig. 4e). (**c**) [^1^H, ^13^C]-correlation spectrum of methyl-labeled and perdeuterated LptC in complex with unlabeled LptA_m_ with (red) or without (black) 2.1 mg/ml of LPS. (**d**) [^1^H, ^15^N]-correlation spectrum of [^2^H, ^15^N]-labeled LptA_m_ in complex with unlabeled LptC without (green) or with (red) 2.1 mg/ml of LPS. The resonances assigned in the free form of LptA_m_ are indicated by a cross.

LPS transfers *in vivo* and *in vitro* from LptC to LptA in a non-reversible way^11, 19^. In order to obtain insights into the mechanism of transfer from LptC to LptA, we titrated the LptC-LptA_m_ complex with LPS and monitored LptC methyl groups, as well as LptA_m_ NH groups upon interaction of LPS with the complex. LPS causes similar CSP at the N-terminus of LptC in the LptC-LptA_m_ complex than in the LptC dimer (Fig. 7 and Figure S5) suggesting that in the former complex LPS is also binding to the cavity formed at the LptC dimerisation interface. Interestingly additional perturbations are observed at the C-terminus of LptC upon addition of LPS to the LptC-LptA_m_ complex, most significantly around I175 which is located in direct contact with LptA_m_ in the complex (Fig. 7b and c). When LptA_m_ NH groups instead of LptC residues are monitored along the titration of the LptC-LptA_m_ complex by LPS, some resonances are clearly impacted, suggesting that LPS also binds to LptA_m_ in the complex (Fig. 7a). Several of these resonances are unassigned as the N-terminus of LptA_m_, involved in LptC binding, was highly shifted during complex formation (Fig. 4). These resonances affected by LPS interaction nevertheless most probably belong to the interface with LptC.

LptA_m_ mutant is active *in vivo* and is thus capable of binding and transporting LPS. In order to evaluate if it is able to bind to LPS *in vitro*, LptA_m_ was titrated with LPS and its NH groups monitored by NMR. LPS induce significant chemical shifts in LptA_m_, confirming it is able to bind LPS (Figure S5). The CSPs observed on LptA_m_ in the LptC-LptA_m_ complex in presence of LPS occur upon direct binding of LPS to LptA_m_, and not from conformational changes upon binding of LPS to LptC. Taken altogether the CSP observed on LptC C-terminus and on LptA_m_ N-terminus in the LptC-LptA_m_ complex suggest that additionally to the LptC-LptC interface, LPS binding occurs also at the LptC-LptA_m_ interface.

## Discussion

LptC-LptA and the N-terminal region of LptD were proposed to form a periplasmic bridge connecting Gram negative bacterial inner and outer membrane to transport the LPS^21^. This protein bridge, is constituted by one LptC, one LptD and a multimer of LptA of unknown length^22, 23^. Mutagenesis and crystallographic studies of LptA suggest that it can interact with itself through its N- and C-termini to form a head to tail multimeric assembly^18^ with an affinity of 29 μM^26^. In parallel, LptA can interact through its N-terminus with LptC to form an LptC-LptA complex at one of the extremity of the bridge model with an affinity of 4 μM^26^. At the OM LptA contacts via its C-terminal β-sheet the N-terminal periplasmic domain of LptD^16^.

Since the structure of LptC and LptA were only determined separately by X-ray and in the absence of LPS in the crystals, we designed a truncated version of LptA where the last C-terminus β-sheet was removed in order to disrupt the LptA/LptA multimer interface and to study in solution the LptC-LptA complex. SEC-MALLS data and SAXS recorded on LptA_m_ confirm a monomeric behavior for this protein in solution. Interestingly, this monomeric LptA_m_ is still able to support cell growth *in vivo* revealing that the property of LptA to multimerize in solution is not strictly required to accomplish LPS transport to the OM. This result, in complement with the lack of stability observed for the wild type LptA-LptA complex by EPR^26^, supports the idea that a multimeric LptA bridge is not always strongly assembled. The proposed stable bridge, connecting the 21-nm-wide periplasm^30^, could dynamically assemble, potentially depending on the presence of LPS and on the local periplasm width. The length of the periplasmic Lpt machinery formed only with a monomeric LptA, one LptC, and the two periplasmic parts of LptF-LptG and LptD-E complexes can be approximated to 16 nm. The length of this assembly could be sufficient to cover the periplasmic width at least in the narrower region of the periplasmic interstice.

In *E. coli* and in absence of mutation in LptF, assembly of LptC and LptA is essential for the LPS transport^31^ and presents a stronger affinity than the LptA-LptA complex^26^. This complex was proposed to be stabilized by a head-to-tail interaction between the two jellyroll protein structures. Our low resolution structure of the LptC-LptA_m_ complex obtained by a combination of SAXS and NMR data, shows the formation of a symmetrical complex composed by a LptC dimer sandwiched by two monomeric LptA_m_. In this LptC-LptA_m_ complex, LptC adopts a position at the N-terminus of LptA_m_ similar to the LptA/LptA interface in the LptA X-ray oligomer. This result strongly suggests that the β-jellyroll fold found in LptF, LptG, LptC, LptA and the N-terminal region of LptD can act as a “lego” brick that allows the establishment in solution of different intermolecular complexes in a head-to-tail manner. In the absence of LptC membrane anchor, a strong dimerization of this functional LptC construct is observed by NMR with an affinity in the nano-molar range. LptC also remains dimeric in solution upon interaction with LptA_m_, forming thus a symmetrical complex. *In vivo*, one of the LptC monomers might be replaced by the periplasmic part of LptF or LptG that also adopt a β-jellyroll structure^12^. The β-jellyroll fold may also account for the peculiar flexibility of the Lpt transport system. Here, we show that LPS transport can be, at least in part, accomplished by Lpt machines carrying LptA_m_, a protein where the last C-terminus β-sheet is removed. Since LptA_m_ is still proficient in LptC interaction we speculate that the truncated protein can still take contact with the LptDE translocon although with a lower efficiency compared with the wild type protein. This result nicely parallels the finding that cells carrying a C-terminally truncated LptC missing the last 53 amino acids are viable^31^. It has been proposed that in these mutants cells the unstable C-terminally truncated LptC protein, stabilized by LptB overexpression, can still be recruited in the Lpt system and interact with LptA to build functional LPS export machineries^31^.

**Figure 8:**
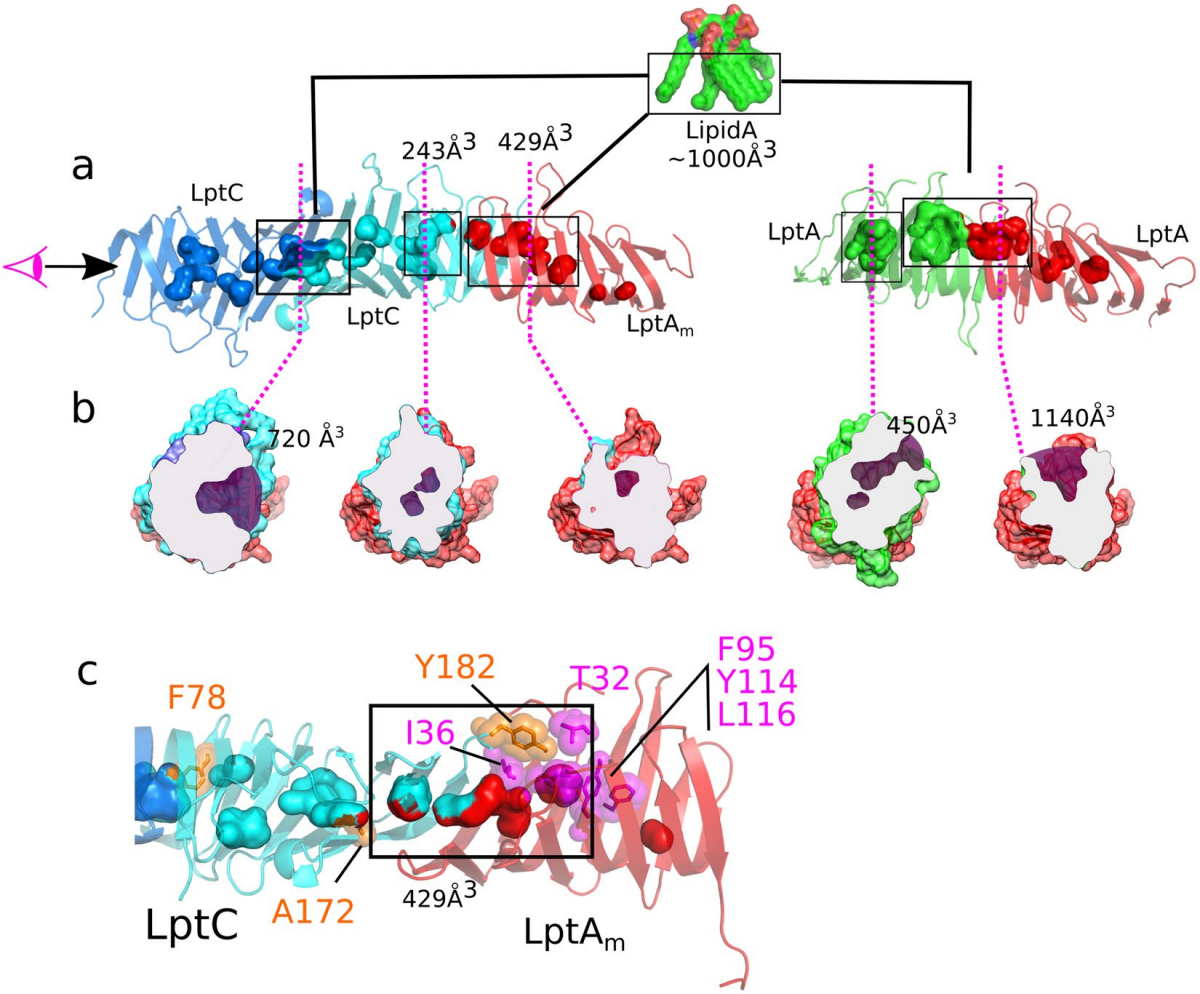
Cavities at protein-protein interfaces are key for interaction of Lpt multimers with LPS. (**a**) Representation of cavities (shown as volume shapes on the ribbon protein structure) at LptC-LptC and LptC-LptA_m_ interface on the model established in this study (left) and at LptA-LptA interface (right, PDB:2R1A chains **b** and **c**). (**b**) Cross-sections of LptC-LptA_m_ and LptA-LptA dimer. Cavities are colored in purple while the molecule cutting plane is shown as a grey surface. (**c**) LptC-LptA_m_ interface showing cavities identically as in (**a**) but with residues shown to form crosslinks with LPS^11^.

To decipher the transfer of LPS through the periplasm, we studied the interaction of LPS with LptC alone, and with LptC-LptA_m_ complex in solution. *In vivo*, LptC is naturally loaded with LPS by the LptB/F/G complex but it is also able to bind LPS present in solution *in vitro* ^19, 32^. The low solubility of LPS in solution is due to its lipid moiety, and consequently concentration of free LPS is low relative to the protein at NMR concentration. Binding attempts with *E. coli* LPS lacking the O-antigen failed, even when solubilized in detergents. We have thus used the smooth LPS molecule possessing a long hydrophilic O-antigen part, which enhances the water solubility, to observe the interaction. NMR shows that the LptC dimer binds LPS in the cavity that is formed at the head-to-head dimer interface involving the N-terminus of each protein monomer. This cavity with an approximate volume of 720 Å^3^ is ideal to accommodate the lipid part of LPS which occupies about 1000 Å^3^ (Fig. 8). The LptC-LPS model suggests that LPS can be bound with minimal conformational changes in LptC. The complex is stabilized by a series of interaction with few conserved aromatic residues at the closure of the pocket (Y60, Y69, and F78) on top of a few electrostatic interactions between phosphate and carbonyl groups of the LPS and several charged amino acids of the protein (R107 and N105) (Figure S7). Nevertheless the LptC-LPS interaction observed by NMR is not strong, as CSP observed are on a fast timescale with respect to chemical shift suggesting a fast k_off_ of the LPS molecule. LptA_m_ protein was also tested by NMR for binding to LPS and significant CSP are induced by LPS, mostly in the second half of the B-jellyroll (SI-5).

When the LptC-LptA_m_ complex is formed, CSP on a fast timescale are also observed upon LPS binding. LptC N-terminal cavity is still binding LPS but additional perturbations are observed at the LptC-LptA_m_ interface both on LptC and LptA_m_ (Fig. 7). Perturbations at the sites of LptC-LptA_m_ interface were not observed upon LPS binding on the isolated proteins and suggest the presence of an additional binding site. This region is in agreement with the location of photo-crosslinkable aminoacids upon interaction with LPS^11^. In the LptC-LptA_m_ complex, several residues forming crosslinks with LPS delimit a 429 Å^3^ cavity located at the LptC-LptA_m_ interface identified in our model (Fig. 8). In particular Y182 crosslink in LptC clusters at the interface with T32, I36, L116, Y114 crosslinks in LptA. Analysis of the size of this second cavity (Fig. 8) shows a volume of only 429 Å^3^ versus 720 Å^3^ for the LptC-LptC interface. This area will thus have to undergo significant opening to adapt to the LPS molecule. On the contrary, in the LptA-LptA complex structures solved by X-ray (PDB:2R1A), a third cavity of 1140 Å^3^ is present at the molecular interface, which could be used to receive the lipidic part of the LPS during the transfer.

Altogether the determination of a LptC-LptA model, LPS binding experiments and analysis of structures suggest that the three intermolecular cavities LptC-LptC, LptC-LptA and LptA-LptA are important sites for LPS binding. These sites, present only when specific complexes between β-jellyroll proteins are assembled could be essential to understand the mechanism of the LPS flow through the Lpt system. In particular, the size variation of the hydrophobic groove along the Lpt bridge, as well as its poor accessibility in some regions (Fig. 8b), is not in favor of a transport acting linearly along a continuous rail^21^ but more a “jump” model where LPS molecules might slide between intermolecular cavities.

## Methods

### Strains, Plasmids, Primers and bacterial growth conditions

*E. coli* bacterial strains and plasmids used in this work are listed in Tables S2 and S3, respectively. A brief outline of plasmids construction by standard techniques has been reported in Table S2. Oligonucleotides used in plasmid constructions are listed in Table S4. All cloned DNA regions obtained by PCR were sequenced to rule out the presence of mutations.

*lptAΔ*_*160-185*_ *lptB* DNA region was obtained by two-step PCR using the external primers AP55-AP35 and, as templates, the *lptA* region encoding for aminoacids 1–159 of LptA (LptA_m_) was PCR-amplified from pGS105 using AP55-AP295 primers, and the *lptB* gene was obtained by PCR amplification of pGS105 DNA with oligonucleotide pair AP296-AP92. The DNA fragment obtained by two-step PCR was digested with *EcoRI* and *XbaI* and cloned in the corresponding sites of pGS100 plasmid, giving pGS105_Δ160-185_. Finally, *lptAΔ*_*160-185*_ *lptB* operon was excised from plasmid pGS105_Δ160-185_ and subcloned into *EcoRI*-*HindIII* sites of pWSK29.

Bacteria were grown at 30 °C in LD^33^, or M9 minimal medium supplemented with 0.2% glucose as a carbon source^34^, with the addition of 0.2% arabinose, 100 μg/ml ampicillin, and 25 μg/ml kanamycin when required. Solid media were as described above with 1% (w/v) agar.

### LPS extraction and purification

Strain selected for LPS production was *Escherichia coli* O157:H7 (Sakai) producing the full length “smooth” LPS molecule comprising lipid A, core oligosaccharide and O antigen. ^13^C-labeling was performed by growing the indicated strain in modified M9 medium (Table S5) with ^13^C glucose as the sole carbon source. Cells were harvested from 1 liter of bacterial culture grown to OD_600_ of 0.8. The lyophilized bacterial pellet was washed several times with distilled water, ethanol and acetone followed by several ultracentrifugation steps (45,000 rpm, 4 °C, 16 h), in order to remove contaminants. Cells were extracted by hot phenol/water extraction^35^. Water and phenol phases were both dialyzed and lyophilized. After inspection by SDS-PAGE, an enzymatic treatment to remove proteins and nucleic acids was executed (37 °C and 56 °C, 5 h and 16 h respectively), followed by a dialysis step. The SDS-PAGE executed on both purified water and phenol phases highlighted the presence of LPS only in the water phase. To further purify the LPS material from a neutral polysaccharide capsular fraction, an ultracentrifugation step (45,000 rpm, 4 °C, 16 h) was executed as well.

### LptA_m_ functionality assay in solid and liquid media

The LptA_m_ functionality was assessed by complementation analysis of *araBp lptA-lptB* conditional expression mutant. The analysis was performed at 30 °C using stationary-phase cultures of FL907(*araBp lptA-lptB*) transformed with suitable pWSK29-based plasmids grown in LD media supplemented with arabinose (0.2%) and ampicillin (100 μg/ml). Cultures were serially diluted in LD in microtiter plates and replica plated on LD agar plates supplemented with ampicillin with and without arabinose and incubated overnight [16–18 h]. To assess the effect of LptA_m_ expression on growth in liquid media, bacterial cultures of the above mentioned strains grown at 30 °C in LD supplemented with 0.2% arabinose and ampicillin (100 μg/ml) were harvested by centrifugation after they had reached an OD_600_ = 0.2, washed in LD, and diluted to OD_600_ of 0.0006 in fresh medium supplemented with ampicillin (100 μg/ml) with or without arabinose. Growth was monitored by measuring OD_600_.

Determination of LptA, and LptA_m_ levels. LptA, and LptA_m_ levels were assessed in FL907 co-expressing wild type or truncated LptA proteins with LptB by Western blot analysis using polyclonal antibodies raised in rabbit against LptA. FL907 cells were grown as described above. Samples for protein analysis were collected 300 min after the shift to non-permissive conditions, centrifuged (16,000 *g*, 5 min), and pellets were resuspended in a volume (in ml) of SDS sample buffer equal to 1/12 of the total OD of the sample. Samples were boiled for 5 min, and equal volumes (20 μl) were analyzed by 12.5% polyacrylamide-SDS gel electrophoresis. The wild type AM604 strain was grown in LD up to OD_600_ of 0.6 and samples for protein analysis were prepared as described above. Proteins were transferred onto nitrocellulose membranes (GE Healthcare), and Western blot analysis was performed as previously described^7^. Polyclonal sera raised against LptA (GenScript Corporation) were used as primary antibody at a dilution of 1:1000.

### Protein production and purification

A LptC pQESH (QIAGEN) plasmid containing an N-terminal His-Tag and lacking the first 23 residues transmembrane domain was used to express *E. coli* LptC^36^. M15 [prep4] cells (QIAGEN) were transformed with the plasmid and grown in LB or M9 minimal medium at 30 °C, induced at an OD_600_ of 0.6 with 0.5 mM IPTG, and left overnight at 20 °C with 25 µg/ml kanamycin and 100 µg/ml ampicillin. Cells were broken by sonication in buffer A (50 mM Na_2_HPO_4_, 300 mM NaCl, 5 mM imidazole, 10% Glycerol, pH 8), centrifuged and the soluble fraction was injected on a 1 ml HiTrap column (GE Healthcare). A gradient of Buffer A supplemented with 300 mM imidazole eluted the protein, which was then injected onto a S75 26/600 GL SEC column in buffer B (50 mM sodium phosphate,150 mM NaCl, pH 8).

LptA_m_ coding for residues 28–159 followed by a SGRVEHHHHHH TAG in a pET21b vector was transformed into BL21(DE3) and grown in a modified M9 minimal medium (Table S5) at 30 °C, induced at an OD_600_ of 0.6 with 0.5 mM IPTG, and left overnight at 20 °C with 100 µg/ml ampicillin^23^. Purification is as described for LptC except that the HiTrap Elution buffer contains 500 mM imidazole.

^13^C- and ^15^N-labeling, as well as perdeuteration was performed as described^37^ with standard M9 medium for LptC. Methyl labeling was achieved according to standard protocols^38–40^ using with NMRbio precursors (http://www.nmr-bio.com/).

### NMR spectroscopy

NMR experiments were recorded on Bruker 600, 700, 850 and 950 MHz spectrometers equipped with triple ^1^H, ^13^C, ^15^N resonance cryoprobes. Experiments on LptC alone, LptA_m_ alone and the LptC-LptA_m_ complex were recorded at 45 °C, 25 °C, and 35 °C, respectively, in buffer B with 5% D_2_O unless stated. Main chain assignment of LptC and LptA_m_ was done on [^2^H, ^13^C, ^15^N]- labeled samples using 2D-[^1^H, ^15^N,]-BEST-TROSY, 3D-BEST-TROSY-HNCACB, 3BEST-TROSY-HN(CO)CACB, 3D-BEST-TROSY-HN(CO), and 3D-BEST-TROSY-HN(CA)CO experiments^41^. LptC backbone assignment was determined first in a 50 mM sodium phosphate,150 mM NaCl, pH 6 buffer and the assignment transferred to the buffer B conditions. Methyl groups were assigned using a 3D-((H)C-TOCSY-C-TOCSY-(C)H) experiment on a [^2^H, ^13^C, ^15^N, ^1^H-(A^β^I^γ2δ1^L^δ1^V^γ1^)]- sample^40^ and a methyl specific 3D-HMQC-NOESY-HMQC (600 ms of NOESY mixing time) on a [^2^H, ^15^N, ^1^H/^13^C-(A^β^I^δ1^L^δ1^V^γ1^)] sample, all in 100% deuterated buffer. Interaction experiments were followed using 2D-[^1^H, ^15^N]-BEST-TROSY, 2D-[^1^H, ^15^N]-SOFAST-HMQC or 2D-[^1^H, ^13^C]-Methyl-BEST-TROSY experiments.

### SEC-MALLS

Protein samples (40 µl) are injected in buffer B at 25 °C on a Superdex S200(10/300GL) connected to an HPLC system with on-line Multi Angle light scattering (DynaPro Nanostar), refraction index (Optilab rex) and Optical density detectors (SPD-M20A). Data analysis is performed with ASTRA 5.4.3.20 software (WYATT) using theoretical dn/dc values of 0.188 and 0.187 ml/g and massic extinction coefficients of 1206 (theoretical protparam (http://web.expasy.org/protparam/) and 466 (obtained experimentally by aminoacid analysis) ml.g^−1^.cm^−1^ for LptC and LptA_m_, respectively. Two-component analysis with the protein conjugate method was used for the LptC-LptA_m_ complex mass and stoichiometry determination.

### Small Angle X-ray Scattering

SAXS data were collected on beamline BM29 from the European Synchrotron Radiation Facility in Grenoble (France) on samples at concentrations of 1.6 and 2.4 mg/ml for LptA_m_, 1, 2 and 3 mg/ml for LptC and 3.6 and 5.4 mg/ml of a LptA_m_:LptC 1:1 molar ratio in the same buffer as for the NMR experiments, at 20 °C, and supplemented with 0.5% w/v sucrose to avoid radiation damage. Ten frames of 1 second each were recorded on each sample, positioned at 2.86 m from a Pilatus detector, at a wavelength of 0.99 Å. For each sample, frames were normalized to the intensity of the transmitted beam before being merged. Buffer’s contribution to the scattering was subtracted automatically by ISPYB^42^. Data were, for each sample type, extrapolated to zero dilution using the PRIMUS software from the ATSAS 2.5.1 software package^43^. Radius of gyration, *Rg*, forward scattering intensity, *I(0)*, maximum particle dimension, *D*_*max*_, and distance distribution function, *P(r)*, were calculated with GNOM from the same program suite. Dammif^44^ in slow mode calculated 20 bead-model envelopes from the SAXS data, models were averaged and filtered (with damaver and damfilt, respectively^43^) to obtain the final *ab-initio* envelopes. Dammif was executed with a P2 symmetry for LptC and LptC-LptA_m_ complex. Back- calculated diffusion data were calculated with Crysol^45^ with a modified solvent density to account for the buffer composition and systematic buffer substraction. Superimposition of molecular structures and envelopes was done with supcomb^46^. LptC N- and C- termini and LptA_m_ C-termini and 78–83 loop, missing from their respective crystal structures (3MY2 and 2R1A chain B, respectively) were added with XPLOR-NIH and minimized with simulated annealing protocol. The obtained structures were used in the figures showing the LptA_m_ monomer or LptC dimer in the current study, as well as inputs for the HADDOCK software^47^ used for data-driven docking.

### Data-driven docking (HADDOCK)

LptC-LptA_m_ docking was performed for half of the complex (1xLPtC + 1xLptA_m_) to speed-up calculations and the full complex was rebuilt according to the LptC dimer symmetry. Residues used to established Ambiguous Interaction Restraints (AIR) in HADDOCK were LptC residues from methyl titration experiments (A119, L151, L174, I175, V178, I184) with a Relative Surface Accessibility (RSA) superior to 10% (as calculated with the NACCESS software). LptA_m_ residues for which NH resonances could not be followed upon LptC interaction and with a RSA superior to 40% were considered active, as well as residues 32, 40 and 115, which experience high CSP. HADDOCK 2.2 online (http://haddock.science.uu.nl/services/HADDOCK2.2/) was run with 2000 rigid body structures, 400 structures refined and water refined, automatic passive residues definition, and clustering by default.

LPS docking onto the LptC dimer or the LptC-LptA_m_ complex was also performed with HADDOCK. The chemical structure of the LPS molecule, containing the lipid A and core sugars, was extracted from the TLR4/MD2 complex (PDB 3FXI), and topology and parameter files were generated from the PRODRG software^48^. All of the atoms of the LPS molecule were considered as passive. LptC residues L58, L62, V67, I136, V143, L167, A172 and S59, Y60, Y70, F78, T79, L83, T93 with CSP superior to twice the standard deviation of shifts were selected from [^1^H, ^13^C]-methyl and [^1^H, ^15^N]-correlation experiments, respectively, and were considered as active residues. HADDOCK rigid body first iteration was performed with the inter_rigid parameter set to zero to allow LPS to reach the inside of the LptC cavity. Calculation was performed with 1000 rigid body structures, 200 structures refined and water refined. Clustering was performed with chimera.

### Determination and representation of cavities in the structural models

Cavity volumes were measured with chimera^49^ for enclosed cavities, and with the 3 V software^50^ for open cavities with 5-Å and 1-Å radii for outside and inside spheres, respectively.

## Electronic supplementary material

Supplementary information

## References

[1] Silhavy, T. J., Kahne, D. & Walker, S. The bacterial cell envelope. *Cold Spring Harbor perspectives in biology* **2** (2010). 10.1101/cshperspect.a000414 PMC2857177 20452953

[2] Whitfield C, Trent MS (2014). Biosynthesis and export of bacterial lipopolysaccharides. Annu. Rev. Biochem..

[3] Nikaido H (2003). Molecular basis of bacterial outer membrane permeability revisited. Microbiol. Mol. Biol. Rev..

[4] Polissi A, Sperandeo P (2014). The lipopolysaccharide export pathway in Escherichia coli: Structure, organization and regulated assembly of the Lpt machinery. Mar. Drugs.

[5] Chng SS, Gronenberg LS, Kahne D (2010). Proteins required for lipopolysaccharide assembly in escherichia coli form a transenvelope complex. Biochemistry.

[6] Sperandeo, P., Martorana, A. M. & Polissi, A. Lipopolysaccharide biogenesis and transport at the outer membrane of Gram-negative bacteria. *Biochim. Biophys. Acta - Mol. Cell Biol. Lipids*, doi:10.1016/j.bbalip.2016.10.006 (2016). 10.1016/j.bbalip.2016.10.006 27760389

[7] Sperandeo P (2007). Characterization of lptA and lptB, two essential genes implicated in lipopolysaccharide transport to the outer membrane of Escherichia coli. J. Bacteriol..

[8] Sperandeo P (2008). Functional analysis of the protein machinery required for transport of lipopolysaccharide to the outer membrane of Escherichia coli. J. Bacteriol..

[9] Ruiz N, Gronenberg LS, Kahne D, Silhavy TJ (2008). Identification of two inner-membrane proteins required for the transport of lipopolysaccharide to the outer membrane of Escherichia coli. Proc. Natl. Acad. Sci. USA..

[10] Narita, S. & Tokuda, H. *Biochemical characterization of an ABC transporter LptBFGC complex required for the outer membrane sorting of lipopolysaccharides*. *FEBS Letters* **583** (2009). 10.1016/j.febslet.2009.05.051 19500581

[11] Okuda S, Freinkman E, Kahne D (2012). Cytoplasmic ATP hydrolysis powers transport of lipopolysaccharide across the periplasm in E. coli. Science.

[12] Luo Q (2017). Structural basis for lipopolysaccharide extraction by ABC transporter LptB2FG. Nat. Struct. Mol. Biol..

[13] Braun M, Silhavy TJ (2002). Imp/OstA is required for cell envelope biogenesis in Escherichia coli. Mol. Microbiol..

[14] Bos MP, Tommassen J (2004). Biogenesis of the Gram-negative bacterial outer membrane. Curr. Opin. Microbiol..

[15] Wu T (2006). Identification of a protein complex that assembles lipopolysaccharide in the outer membrane of Escherichia coli. Proc. Natl. Acad. Sci. U. S. A..

[16] Freinkman E, Okuda S, Ruiz N, Kahne D (2012). Regulated assembly of the transenvelope protein complex required for lipopolysaccharide export. Biochemistry.

[17] Villa R (2013). The Escherichia coli lpt transenvelope protein complex for lipopolysaccharide export is assembled via conserved structurally homologous domains. J. Bacteriol..

[18] Suits MDL, Sperandeo P, Dehò G, Polissi A, Jia Z (2008). Novel Structure of the Conserved Gram-Negative Lipopolysaccharide Transport Protein A and Mutagenesis Analysis. J. Mol. Biol..

[19] Tran AX, Dong C, Whitfield C (2010). Structure and functional analysis of LptC, a conserved membrane protein involved in the lipopolysaccharide export pathway in Escherichia coli. J. Biol. Chem..

[20] Qiao S, Luo Q, Zhao Y, Zhang XC, Huang Y (2014). Structural basis for lipopolysaccharide insertion in the bacterial outer membrane. Nature.

[21] Okuda S, Sherman DJ, Silhavy TJ, Ruiz N, Kahne D (2016). Lipopolysaccharide transport and assembly at the outer membrane: the PEZ model. Nat. Rev. Microbiol..

[22] Merten JA, Schultz KM, Klug CS (2012). Concentration-dependent oligomerization and oligomeric arrangement of LptA. Protein Sci..

[23] Santambrogio C (2013). LptA assembles into rod-like oligomers involving disorder-to-order transitions. J. Am. Soc. Mass Spectrom..

[24] Srinivas N (2010). Peptidomimetic Antibiotics Target Outer-Membrane Biogenesis in Pseudomonas aeruginosa. Science (80-.)..

[25] Werneburg M (2012). Inhibition of lipopolysaccharide transport to the outer membrane in Pseudomonas aeruginosa by peptidomimetic antibiotics. Chembiochem.

[26] Schultz KM, Feix JB, Klug CS (2013). Disruption of LptA oligomerization and affinity of the LptA-LptC interaction. Protein Sci..

[27] Dominguez C, Boelens R, Bonvin AMJJ (2003). HADDOCK: a protein-protein docking approach based on biochemical or biophysical information. J. Am. Chem. Soc..

[28] Karaca E, Bonvin AMJJ (2013). Advances in integrative modeling of biomolecular complexes. Methods.

[29] Albright S, Agrawal P, Jain NU (2009). NMR spectral mapping of Lipid A molecular patterns affected by interaction with the innate immune receptor CD14. Biochem. Biophys. Res. Commun..

[30] Matias VRF, Al-Amoudi A, Dubochet J, Beveridge TJ (2003). Cryo-transmission electron microscopy of frozen-hydrated sections of Escherichia coli and Pseudomonas aeruginosa. J. Bacteriol..

[31] Martorana AM (2016). Functional interaction between the cytoplasmic ABC protein LptB and the inner membrane LptC protein, components of the lipopolysaccharide transport machinery in Escherichia coli. J. Bacteriol..

[32] Sestito SE (2014). Functional characterization of E. coli LptC: Interaction with LPS and a synthetic ligand. ChemBioChem.

[33] Ghisotti D (1992). Genetic analysis of the immunity region of phage‐plasmid P4. Mol. Microbiol..

[34] Kunz DA, Chapman PJ (1981). Catabolism of pseudocumene and 3-ethyltoluene by Pseudomonas putida (arvilla) mt-2: Evidence for new functions of the TOL (pWWO) plasmid. J. Bacteriol..

[35] Galanos C, Lüderitz O, Westphal O (1969). A New Method for the Extraction of R Lipopolysaccharides. Eur. J. Biochem..

[36] Sperandeo P (2011). New insights into the Lpt machinery for lipopolysaccharide transport to the cell surface: LptA-LptC interaction and LptA stability as sensors of a properly assembled transenvelope complex. J. Bacteriol..

[37] Jean NL (2014). Elongated structure of the outer-membrane activator of peptidoglycan synthesis LpoA: Implications for PBP1A stimulation. Structure.

[38] Ayala I, Sounier R, Usé N, Gans P, Boisbouvier J (2009). An efficient protocol for the complete incorporation of methyl-protonated alanine in perdeuterated protein. J. Biomol. NMR.

[39] Mas G, Crublet E, Hamelin O, Gans P, Boisbouvier J (2013). Specific labeling and assignment strategies of valine methyl groups for NMR studies of high molecular weight proteins. J. Biomol. NMR.

[40] Kerfah R, Hamelin O, Boisbouvier J, Marion D (2015). CH3-specific NMR assignment of alanine, isoleucine, leucine and valine methyl groups in high molecular weight proteins using a single sample. J. Biomol. NMR.

[41] Lescop E, Kern T, Brutscher B (2010). Guidelines for the use of band-selective radiofrequency pulses in hetero-nuclear NMR: Example of longitudinal-relaxation-enhanced BEST-type 1H-15N correlation experiments. J. Magn. Reson..

[42] Antolinos DM (2015). A. *et al*. ISPyB for BioSAXS, the gateway to user autonomy in solution scattering experiments. Acta Crystallogr. Sect. D Biol. Crystallogr..

[43] Petoukhov MV (2012). New developments in the ATSAS program package for small-angle scattering data analysis. J. Appl. Crystallogr..

[44] Franke D, Svergun DI (2009). DAMMIF, a program for rapid ab-initio shape determination in small-angle scattering. J. Appl. Crystallogr..

[45] Svergun D, Barberato C, Koch MH (1995). CRYSOL - A program to evaluate X-ray solution scattering of biological macromolecules from atomic coordinates. J. Appl. Crystallogr..

[46] Kozin MB, Svergun DI (2001). Automated matching of high- and low-resolution structural models. J. Appl. Crystallogr..

[47] Van Zundert GCP (2016). The HADDOCK2.2 Web Server: User-Friendly Integrative Modeling of Biomolecular Complexes. J. Mol. Biol..

[48] Schüttelkopf AW, Van Aalten DMF (2004). PRODRG: a tool for high-throughput crystallography of protein-ligand complexes. Acta Crystallogr. D. Biol. Crystallogr..

[49] Pettersen EF (2004). UCSF Chimera—A Visualization System for Exploratory Research and Analysis. J Comput Chem.

[50] Voss NR, Gerstein M (2010). 3V: Cavity, channel and cleft volume calculator and extractor. Nucleic Acids Res..

[51] Michino H (1999). Massive outbreak of Escherichia coli O157:H7 infection in schoolchildren in Sakai City, Japan, associated with consumption of white radish sprouts. Am. J. Epidemiol..

[52] Miyashita A (2012). Lipopolysaccharide O-antigen of enterohemorrhagic Escherichia coli O157:H7 is required for killing both insects and mammals. FEMS Microbiol. Lett..

