# Supplementary material for: Interaction of lipopolysaccharides at intermolecular sites of the periplasmic Lpt transport assembly
Source: Sci Rep. 2017 Aug 29;7:9715. doi: 10.1038/s41598-017-10136-0 (PMC5575297; doi:10.1038/s41598-017-10136-0)
Supplement: Supplementary file 1 — Supplementary information [file 41598_2017_10136_MOESM1_ESM.pdf]

SUPPLEMENTARY INFORMATION

Interaction of lipopolysaccharides at intermolecular sites of the periplasmic Lpt transport assembly.

Cedric Laguri[1,2,3,*], Paola Sperandeo[4], Kevin Pounot[1,2,3], Isabel Ayala[1,2,3], Alba Silipo[5], Catherine M. Bougault[1,2,3], Antonio Molinaro[5], Alessandra Polissi[4,*] and Jean-Pierre Simorre[1,2,3]

[1] Université Grenoble Alpes, Institut de Biologie Structurale, 71 avenue des Martyrs – CS10090, 38044 Grenoble cedex 9, France

[2] CEA, DSV, Institut de Biologie Structurale, 71 avenue des Martyrs – CS10090, 38044 Grenoble cedex 9, France

[3] CNRS, Institut de Biologie Structurale, 71 avenue des Martyrs – CS10090, 38044 Grenoble cedex 9, France

[4] University of Milano, Department of Pharmacological and Biomolecular Sciences, Via Balzaretti 9, Milano, Italy

[5] University of Naples Federico II, Department of Chemical Sciences, via cinthia 4, Napoli, Italy

[*] **Correspondence: cedric.laguri@ibs.fr (C. L.) and alessandra.polissi@unimi.it (A. P.)**

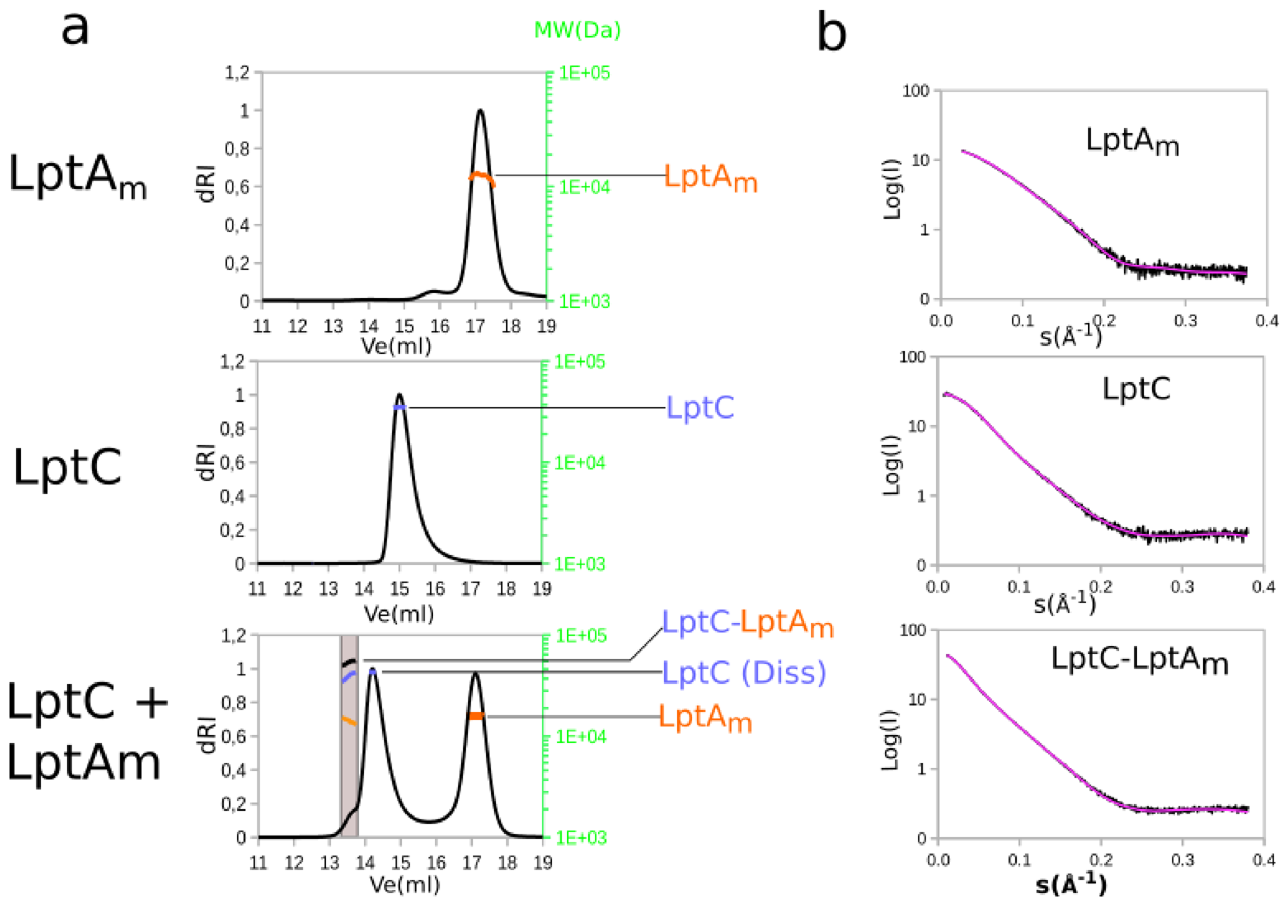

Figure S1

SEC MALLS AND SAXS of LptA$_m$, LptC and LptC-LptA$_m$.

(a) SEC MALLS elution profiles of LptA$_m$, LptC and 1:2 LptC:LptA$_m$ mixture in Buffer B. 40 µl of 100 µM LptA$_m$, 100 µM LptC or a mixture of 200 µM LptC:400 µM LptA$_m$ were injected on a Superdex S200 column. LptA$_m$ is eluted mostly as a monomeric species while LptC is dimeric. Upon injection of the mixture containing both proteins an additional species of 54 kDa appears (grey area) corresponding to a LptC-LptA$_m$ complex of 2:1 stoechiometry. LptC (marked as LptC Diss) is eluted significantly earlier in presence of LptA$_m$ suggesting that it formed a complex that dissociated in the course of the gel filtration. The curve corresponds to the differential refractive index according to the scale on the left and the colored curve corresponds to the molecular weight read according to the scale on the right. **(b)** SAXS of LptA$_m$, LptC and LptC:LptA$_m$ 1:1 mixture**.** The scattering data is shown in black and the back-calculated curve originating from the best NSD (Normal Space Displacement) envelope calculated with Dammif is represented in magenta.

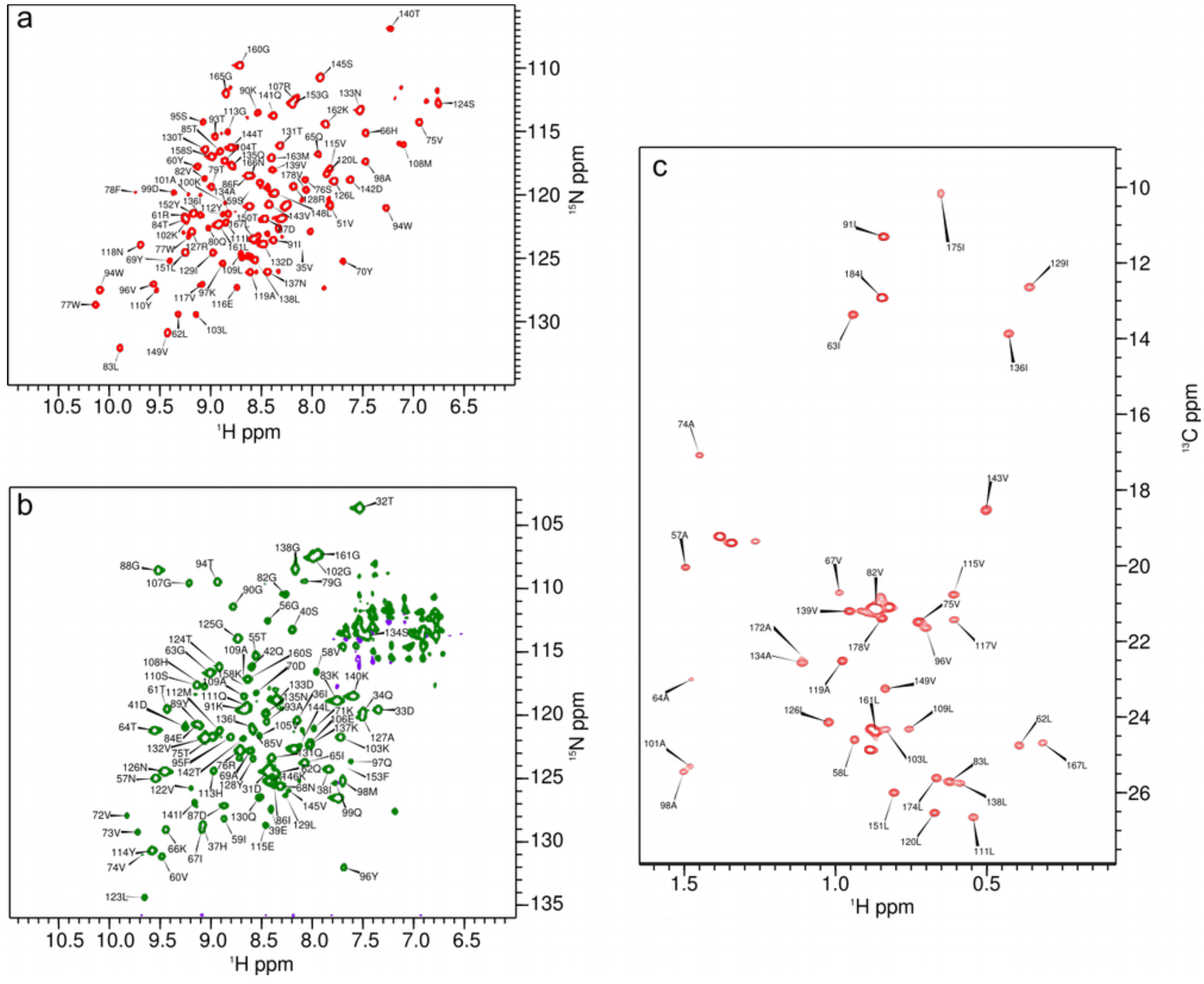

Figure S2

Assigned NMR spectra of LptC and LptA$_{m}$.

(a) 2D $[^{1}H,^{15}N]$-BEST-TROSY of $[^{2}H,^{15}N]$-labeled LptC, **(b)** 2D $[^{1}H,^{15}N]$-BEST-TROSY of $[^{2}H,^{15}N]$-labeled LptA$_{m}$ and **(c)** methyl selective $[^{1}H,^{13}C]$-BEST-TROSY-HMQC of $[^{2}H,^{15}N,\ ^{13}C/^{1}H\text{-}(A^{\beta}I^{\delta 1}L^{\delta 1}V^{\gamma 1})]$-LptC

a

Cluster	Haddock	sd	SAXS χ^2	sd	Had+SAXS	NSD Å	sd	rmsd Å	sd	N_{struc}	$E_{vdw\ kcal/mole}$	sd	$E_{elec\ kcal/mole}$	sd	$E_{air\ kcal/mole}$	sd	$B_{uried}\ S_{urface}$ Å^2	sd
1	**-113**	10	**11**	2	**53**	**1.79**	0.01	1.4	0.9	37	-80	6	-229	58	133	48	2019	178
2	**-94**	5	**15**	2	**98**	**1.95**	0.04	2.8	1.7	38	-58	9	-215	33	111	44	1536	298
3	**-94**	15	**115**	36	**441**	**3.84**	0.10	3.5	1.8	8	-41	3	-338	78	98	28	1387	175
4	**-88**	16	**18**	6	**122**	**2.30**	0.15	5.6	4.2	10	-50	5	-220	62	64	46	1319	209

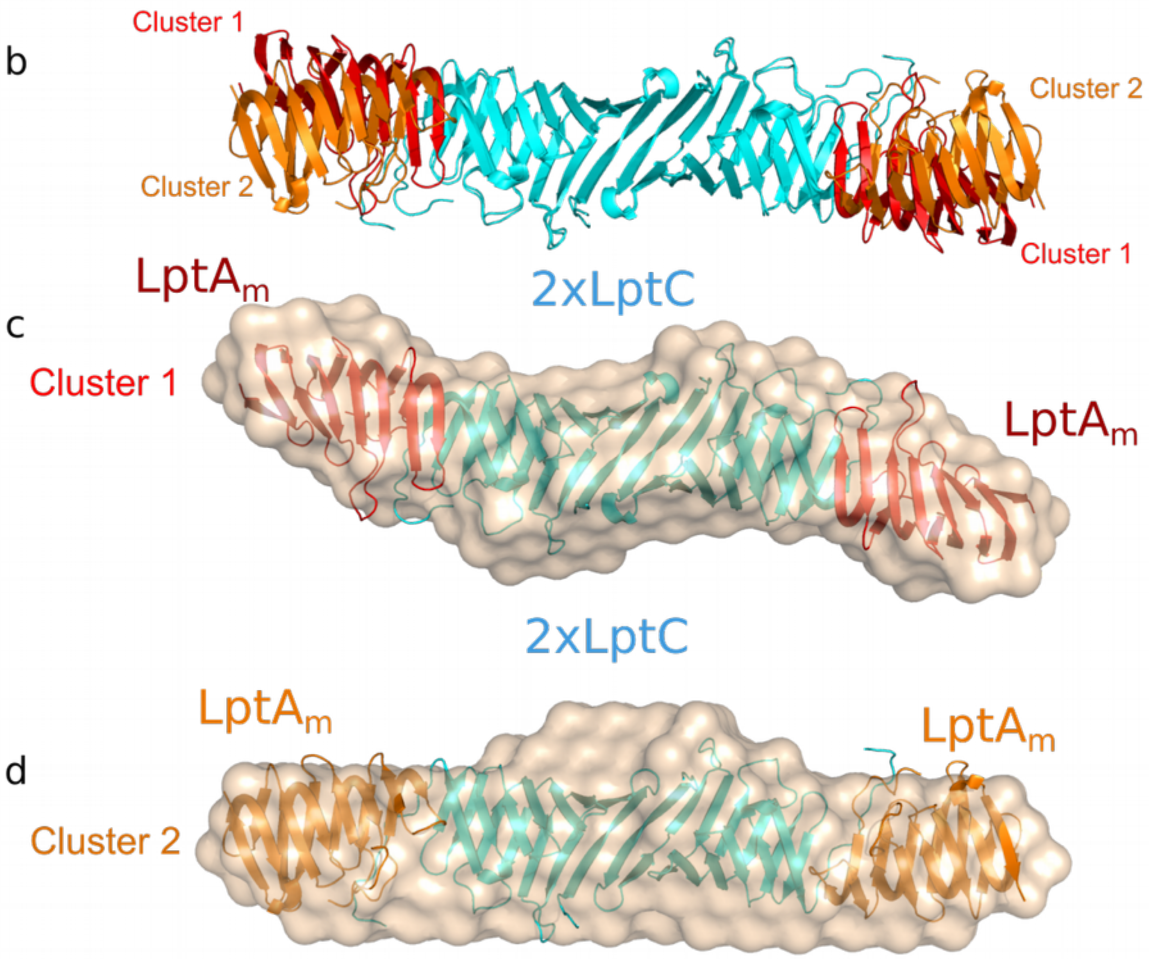

Figure S3

Combined NMR/SAXS HADDOCK model of LptC-LptA$_m$ complexes.

(**a**) HADDOCK statistics and SAXS scores of the different ensembles of solutions obtained with HADDOCK. (sd, standard deviation). (**b**) Ribbon representation of the best energy structures of clusters 1 and 2 with LptA$_m$ colored in red and orange, respectively. Structures are aligned for the LptC backbone. (**c**) Fit of the best structure of cluster 1 inside the SAXS calculated envelope of the complex. (**d**) Fit of the best structure of cluster 2 inside the SAXS calculated envelope of the complex. The LptC orientation is identical in panels b through d.

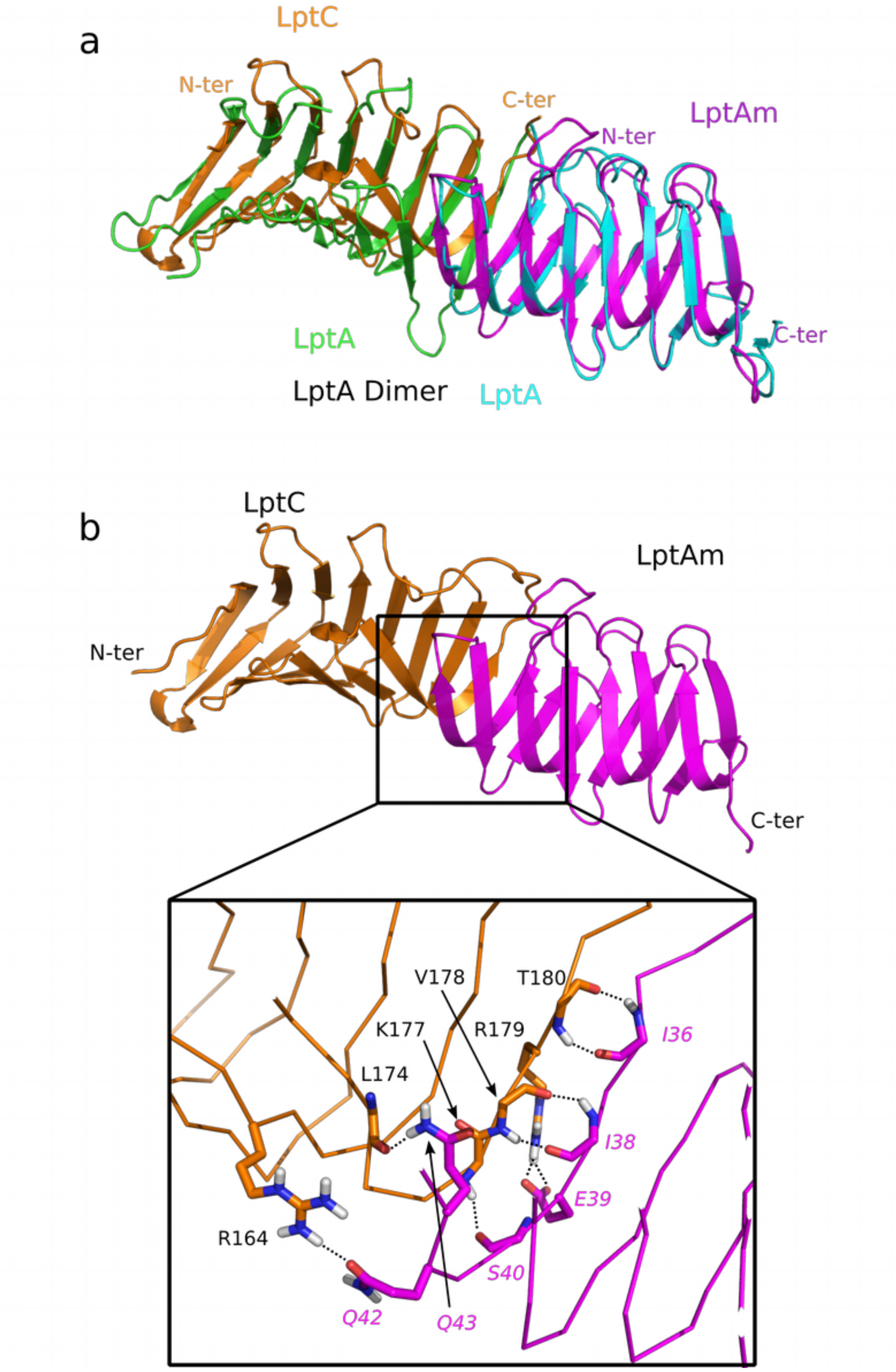

Figure S4

Detailed view of the LptC-LptA$_m$ model and comparison with the LptA-LptA oligomer

(**a**) LptA dimer (PDB 2R1A, chains B and C) was superimposed on LptA$_m$ in the LptC-LptA$_m$ complex.

(**b**) Detailed view of the LptC-LptA$_m$ contacts found in HADDOCK models. Contacts found by Ligplus(Laskowski and Swindells, 2011) in a majority of HADDOCK models are represented.

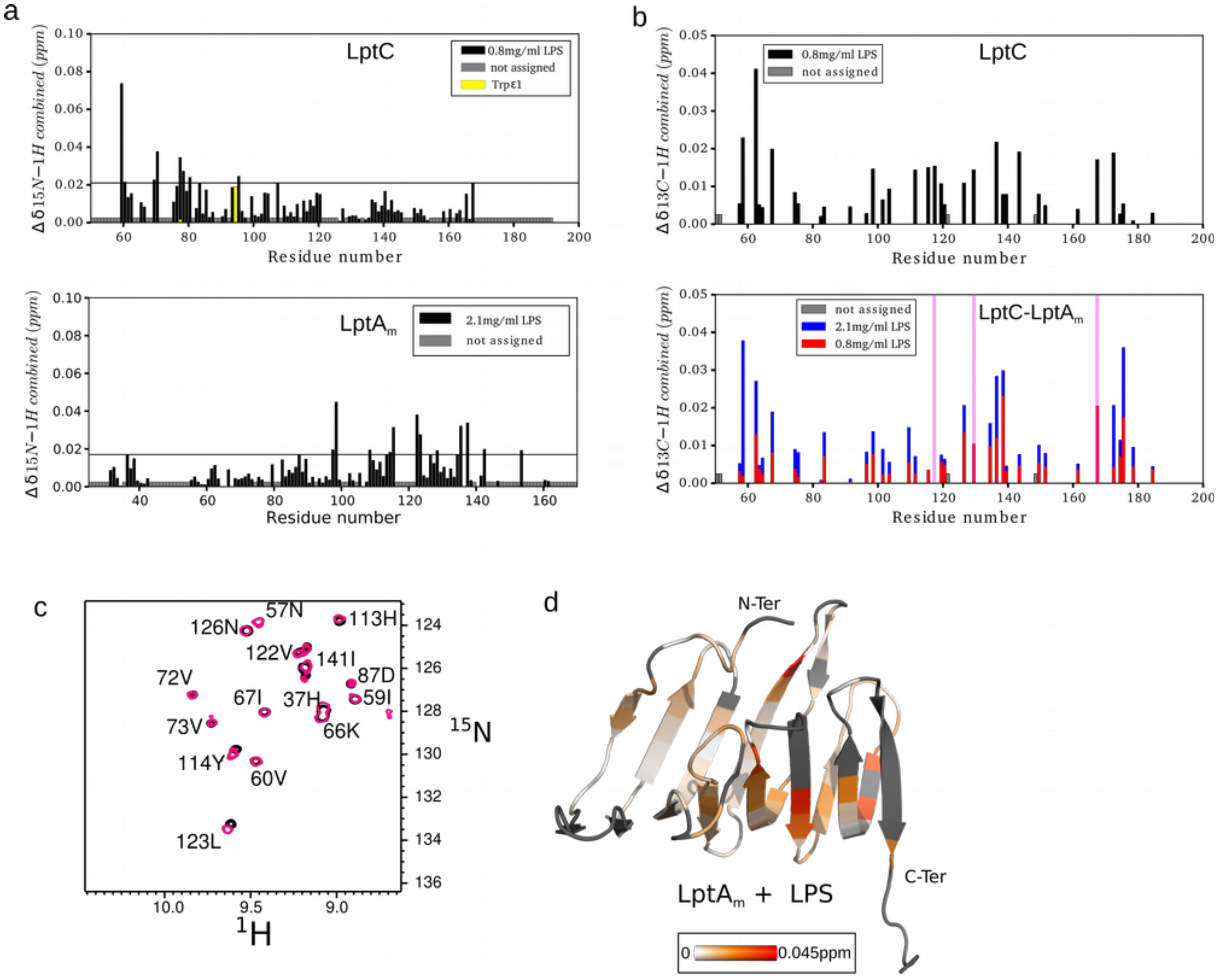

Figure S5

Interaction of LptC, LptA$_m$ and LptC-LptA$_m$ complex with LPS.

(**a**) CSP of LptC and LptA$_m$ NH groups in presence of 0.8 and 2.1 mg/ml of LPS respectively. The horizontal bar represents the value of twice the standard deviation of all CSPs (**b**) CSP of LptC methyls groups upon addition of 0.8 mg/ml LPS when in the LptC dimer alone (black boxes) or when in the complex with LptA$_m$ for two LPS concentrations, 0.8 mg/ml and 2.1 mg/ml (red and blue, respectively). Resonances from residues highlighted in magenta broaden upon LPS addition until they cannot be detected anymore. (c) [^{1}H,^{15}N] correlation spectrum of [^{1}H,^{15}N]LptA$_m$ in absence (black) or presence (magenta) of 2.1 mg/ml of LPS. (d) Representation of ^{1}H and ^{15}N combined CSP induced by LPS (2.1 mg/ml) on LptA$_m$ cartoon. Gradient colors used for the display of the CSP values are shown at the bottom part of the panel.Combined heteronuclear chemical shifts

are expressed according to the expression $\delta Hz=\sqrt{(\delta 1Hppm)^2+(\delta Xppm*\gamma X/\gamma 1H)^2}$ with X being either ^{13}C or ^{15}N and γ the gyromagnetic ratio.

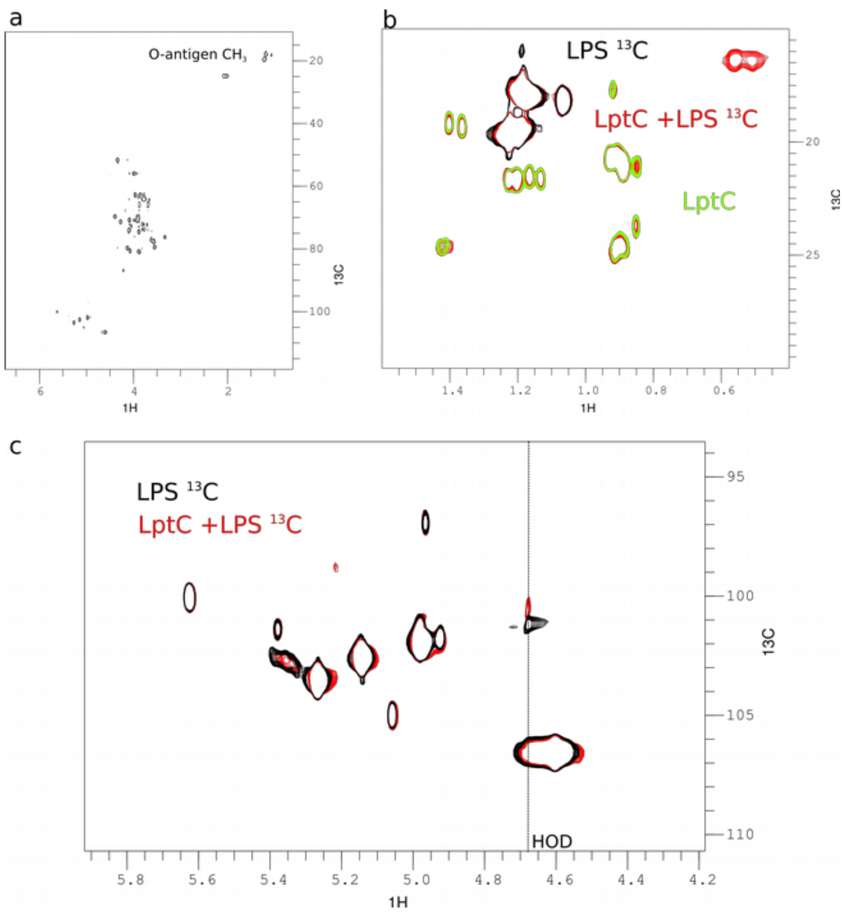

Figure S6.

Impact of interaction of LptC dimer on LPS NMR signature in solution

(**a**) [^{1}H, ^{13}C]-correlation spectrum of 2.1 mg/ml [^{13}C, ^{15}N]-labeled LPS. (**b**) Enlarged view on the methyl area of this spectrum in the absence (black) and in the presence (red) of 400 µM of LptC. Two new peaks belonging to LPS appear at $\delta^{13}C$ = 16.3 ppm, a resonance frequency that is characteristic of the methyl groups at the end of the lipid chain of LPS. In solution, LPS form micelles and larger aggregates(Yu et al., 2006) and here the lipids methyl groups are absent from the NMR spectra in absence of LptC. For reference, the spectrum of 400 µM of LptC is overlaid (green) showing LptC signals in carbon natural abundance. (**c**) Enlarged view of the anomeric area of the [^{1}H, ^{13}C]-correlation spectrum of LPS in the absence (black) and in the presence (red) of 400 µM of LptC.

a

Haddock	sd	rmsd Å	sd	N_{struc}	E_{vdw} kcal/mol	sd	E_{elec} kcal/mol	sd	E_{air} kcal/mol	sd	B_{uried} S_{urface} $Å^2$	sd
-83	15	0.7	0.4	6	-59	10	-53	17	348	110	2687	180

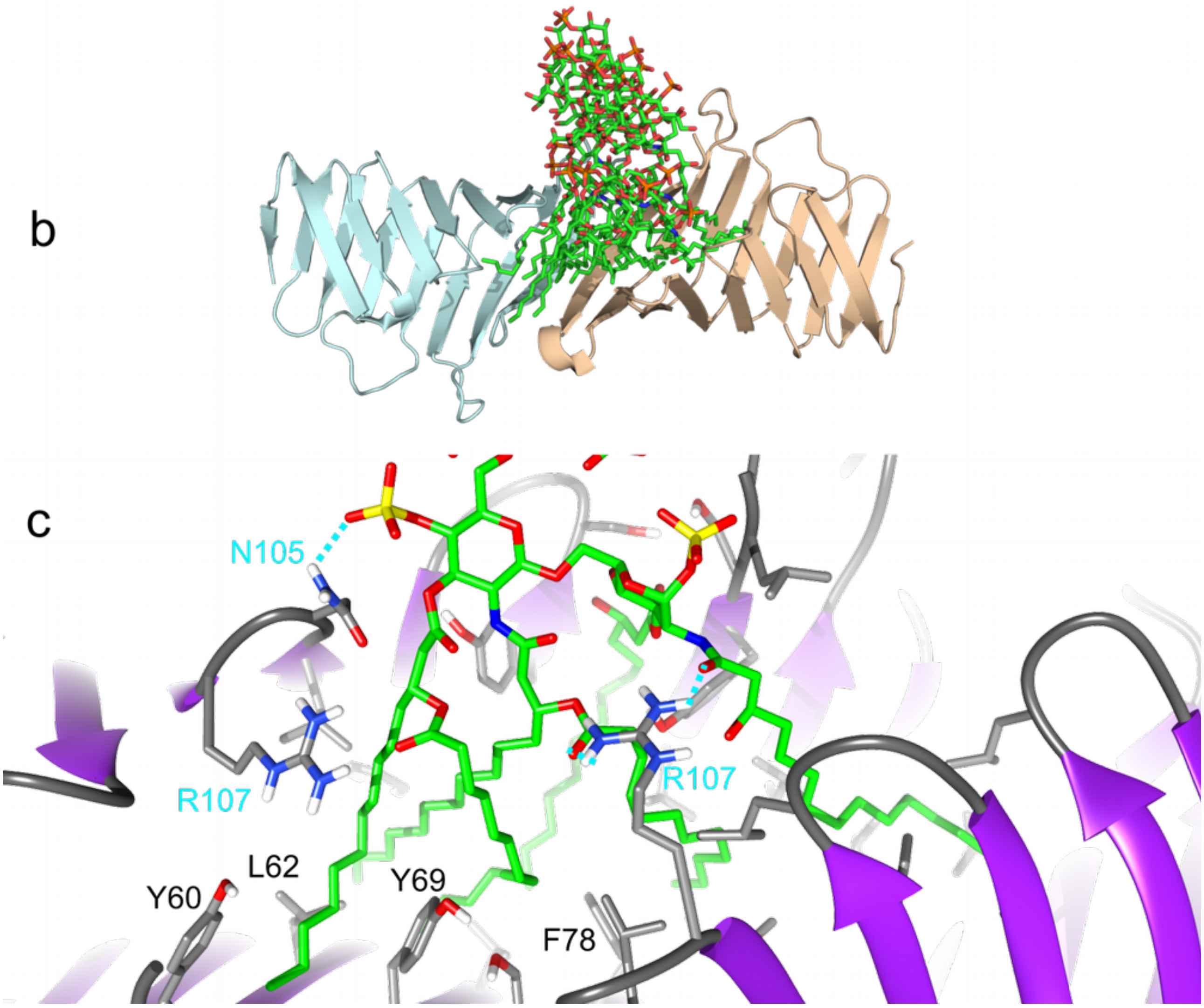

Figure S7

HADDOCK model of LptC in complex with LPS.

(**a**) HADDOCK statistics of the best 4 structures of LptC in complex with LPS. (**b**) Overlaid LPS structures of the 4 best models after alignment on LptC structure. Only the cartoon of the best LptC complex is shown for clarity. (**c**) Details of the LptC-LPS interaction. The lipid chains of LPS insert into the LptC cavity and interact with a number of hydrophobic aminoacids (black labels) in the cavity including several aromatic residues. At the cavity opening, two aminoacids N105 and R107 (in cyan) from both monomers are ideally placed to interact with phosphate groups and/or acyl moieties of the lipid A.

Table S1. Biophysical characterization of LptC, $LptA_m$ and LptC-$LptA_m$ complex.

	MW (kDa) (SEC-MALLS)	R_g (nm) (SAXS)	D_{max} (nm) (SAXS)
$LptA_m$	15.9 (27.7 dim)	2.1	6.2
LptC	37.7	3.0	11.3
LptC/$LptA_m$ complex	54 (2:1)	4.4	17.5

Table S2. *E. coli* strains.

Strain	Genotype	Reference
AM604	MC4100 ara^+	(Wu et al., 2006)
BL21(DE3)	F^- *ompT gal dcm lon* $hsdS_B(r_B^- m_B^-)$ (λDE3 *[lacI lacUV5-T7 gene 1 ind1* Sam7 *nin5])*	(Studier and Moffatt, 1986)
O157:H7 str. Sakai (EHEC)	Human isolate from outbreak associated with white radish sprout in Osaka, Japan, 1996	(Michino et al., 1999)
FL907	AM604 Φ*(kan araC araBp-lptA)1*	(Sperandeo et al., 2008)
M15/pREP4	F^- *lac thi mtl*/pREP4	Qiagen
XL1-Blue	F^- $λ^-$ *recA1 endA1 gyrA96 thi-1 hsdR17 supE44 relA1 lac* {F' *proAB, lacIqZ* Δ*M15* Tn*10*(Tet^R)}	Agilent technologies

Table S3. Plasmids

Plasmid	Parental plasmid/replicon	Relevant characters	Construction/Origin
pET-LptA-H	pET21b	*pT7-lptA*-His$_6$; Amp^R	(Suits et al., 2008)
pET-LptA$\Delta_{160-185}$ –H	pET21b	*pT7-lptA*$\Delta_{160-185}$ -His$_6$; Amp^R	This work: *lptA*$\Delta_{160-185}$ allele was PCR amplified from pWSK29-LptA$\Delta_{160-185}$ LptB with AP182 and AP183 and cloned into *BamHI-NotI* sites of pET-LptA-H
pGS100	pGZ119EH	*ptac*-TIR *cat oriV*$_{ColD}$	(Sperandeo et al., 2006)
pGS105	pGS100	*ptac-lptA lptB*; Cam^R	(Sperandeo et al., 2006)
pGS105$\Delta_{160-185}$	pGS105	*ptac- lptA*$\Delta_{160-185}$ *lptB; Cam*^{R}	This work: *lptA*$\Delta_{160-185}$ *lptB* operon was generated by two-step PCR and cloned into *EcoRI-XbaI* sites of pGS100
pWSK29	pBSIISK	pSC101 *ori* f1 *ori lacZa*; Amp^R	(Wang and Kushner, 1991)
pWSK29-LptA LptB	pWSK29	*plac-lptA lptB*; Amp^R	(Santambrogio et al., 2013)
pWSK29-LptA$\Delta_{160-185}$ LptB	pWSK29-LptA LptB	*plac- lptA*$\Delta_{160-185}$ *lptB;* Amp^R	This work*: lptA*$\Delta_{160-185}$ *lptB* operon was excised from plasmid pGS105$\Delta_{160-185}$ and subcloned into *EcoRI-HindIII* sites of pWSK29

Table S4. Oligonucleotides.

Name	Sequence [a]	Notes
AP35	5'-gactagtctagaCTACCCTATCAGAGTCTGAAGTCTTCC-3'	pGS105$\Delta_{160-185}$ construction with AP55, *XbaI*
AP55	5’-cgagaggaattcAACATGAAATTCAAAACAAACAAACTC-3’	Construction of *lptA*$\Delta_{160-185}$ allele for pGS105$\Delta_{160-185}$ with AP295,, *EcoRI*
AP92	5'-CTGCGTTCTGATTTAATCTG-3'	*lptB* amplification for pGS105$\Delta_{160-185}$ with AP296
AP183	5’-cgagatggatccATGAAATTCAAAACAAACAAAC -3’	pET-LptA$\Delta_{160-185}$ -H construction with AP473, *BamHI*
AP295	5’-GCGCTTGCCTTTGTCGCTG-3’	Construction of *lptA*$\Delta_{160-185}$ allele for pGS105$\Delta_{160-185}$ with AP55
AP296	5'-GCGACAAAGGCAAGCGCTAATTCGTTATGGCAAC-3'	Construction of *lptB* allele for pGS105$\Delta_{160-185}$ with AP35
AP473	5’-ctcgacgcggccgcTGCGCTTGCCTTTGTCGCTGA-3’	pET-LptA$\Delta_{160-185}$ -H construction with AP183, *NotI*

[a] Upper case letters, sequence present in the template; lower case letters, additional/modified sequence not present in the template; restriction sites are underlined.

Table S5. Modified minimal medium for $LptA_m$ expression.

NH_4Cl	1g/l	<u>Sock Salts (1 l)</u>	
Na_2HPO4	6 g/l	$CaCO_3$	2g
KH_2PO4	30g/l	$FeSO_4.7H_2O$	4,5 g
NaCl	0.5 g/l	$ZnSO_4.7H_{2O}$	1,44 g
$MgSO_4$	1mM	$MnSO_4.4H_2O$	1,12 g
$CaCl_2$	0,1 mM	$CuSO_4.5\ H_2O$	0,25 g
**Goodies**	1X	$CoSO_4.7\ H_2O$	0,28 g
Glucose	0,2% w/v	H_3BO_3	0,06 g
<u>Goodies 5000X</u>		Fuming HCl	51,3 ml
**Sock salts**	50 ml		
$MgSO_4$ 1M	25 ml		
$FeSO_4.7H_2O$ 37 mM	25ml		

Supplementary References.

Laskowski, R. A. and Swindells, M. B. (2011) LigPlot+: Multiple Ligand–Protein Interaction Diagrams for Drug Discovery, *Journal of Chemical Information and Modeling*. American Chemical Society, 51(10), pp. 2778–2786.

Michino, H., Araki, K., Minami, S., Takaya, S., Sakai, N., Miyazaki, M., Ono, A. and Yanagawa, H. (1999) Massive outbreak of Escherichia coli O157:H7 infection in schoolchildren in Sakai City, Japan, associated with consumption of white radish sprouts., *American journal of epidemiology*, 150(8), pp. 787–96.

Santambrogio, C., Sperandeo, P., Villa, R., Sobott, F., Polissi, A. and Grandori, R. (2013) LptA assembles into rod-like oligomers involving disorder-to-order transitions, *Journal of the American Society for Mass Spectrometry*, 24(10), pp. 1593–1602.

Sperandeo, P., Lau, F. K., Carpentieri, A., De Castro, C., Molinaro, A., Dehò, G., Silhavy, T. J. and Polissi, A. (2008) Functional analysis of the protein machinery required for transport of lipopolysaccharide to the outer membrane of Escherichia coli, *Journal of Bacteriology*, 190(13), pp. 4460–4469.

Sperandeo, P., Pozzi, C., Dehò, G. and Polissi, A. (2006) Non-essential KDO biosynthesis and new essential cell envelope biogenesis genes in the Escherichia coli yrbG-yhbG locus, *Research in Microbiology*, 157(6), pp. 547–558.

Studier, F. W. and Moffatt, B. A. (1986) Use of bacteriophage T7 RNA polymerase to direct selective high-level expression of cloned genes., *Journal of molecular biology*, 189(1), pp. 113–30.

Suits, M. D. L., Sperandeo, P., Dehò, G., Polissi, A. and Jia, Z. (2008) Novel Structure of the Conserved Gram-Negative Lipopolysaccharide Transport Protein A and Mutagenesis Analysis, *Journal of Molecular Biology*, 380(3), pp. 476–488.

Wang, R. F. and Kushner, S. R. (1991) Construction of versatile low-copy-number vectors for cloning, sequencing and gene expression in Escherichia coli., *Gene*, 100, pp. 195–199.

Wu, T., McCandlish, A. C., Gronenberg, L. S., Chng, S.-S., Silhavy, T. J. and Kahne, D. (2006) Identification of a protein complex that assembles lipopolysaccharide in the outer membrane of Escherichia coli., *Proceedings of the National Academy of Sciences of the United States of America*, 103(31), pp. 11754–11759.

Yu, L., Tan, M., Ho, B., Ding, J. L. and Wohland, T. (2006) Determination of critical micelle concentrations and aggregation numbers by fluorescence correlation spectroscopy: Aggregation of a lipopolysaccharide, *Analytica Chimica Acta*, 556(1), pp. 216–225.
